# Progress and challenges in traceless ligand-directed labelling chemistry

**DOI:** 10.1038/s42004-026-02222-0

**Published:** 2026-09-29

**Authors:** Toby Hopkinson, Liam R. Cox, Andrew J. Wilson

**Affiliations:** 1 School of Chemistry, University of Birmingham, Edgbaston, Birmingham, UK; 2 Astbury Centre for Structural Molecular Biology, University of Leeds, Leeds, UK; 3 School of Chemistry, University of Leeds, Leeds, UK

**Keywords:** Chemical modification, Chemical tools

## Abstract

Traceless ligand-directed labelling (t-LDL) of proteins is emerging as a potential strategy for addressing the challenge of studying biology within native cellular contexts. By exploiting proximity-driven reactivity, t-LDL enables the selective transfer of small functional groups to residues distal to canonical binding or active sites, allowing the delivery ligand to dissociate following labelling. This review summarises the development of t-LDL chemistry, with a focus on transfer and catalyst-tethered approaches. It highlights key recent advances that expand the mechanistic and functional scope of t-LDL alongside outstanding challenges and opportunities for further development. Examples of applications in live-cell imaging, proteomics, proximity-induced post-translational modification and signalling rewiring, showcase the growing opportunities of this labelling technology.

## Abstract (web summary)

Understanding and manipulating protein–protein interactions (PPIs) with spatiotemporal precision is crucial for advancing protein function studies. Here, the authors summarise recent advances in traceless ligand-directed labelling (t-LDL), a promising strategy that enables selective, minimally invasive tagging of proteins in native cellular contexts.

## Introduction

The human proteome is predicted to consist of approximately 20,000 proteins^1^; these facilitate, regulate and carry out a wide variety of cellular functions^2–4^. This gives rise to complex networks of protein-protein interactions (PPIs), signalling cascades and metabolic processes that are fundamental to cell biology. Methods for labelling proteins are therefore of critical importance to understanding these biomolecular mechanisms, mapping intricate PPI networks and elucidating disease-relevant pathways^5,6^.

**Fig. 1:**
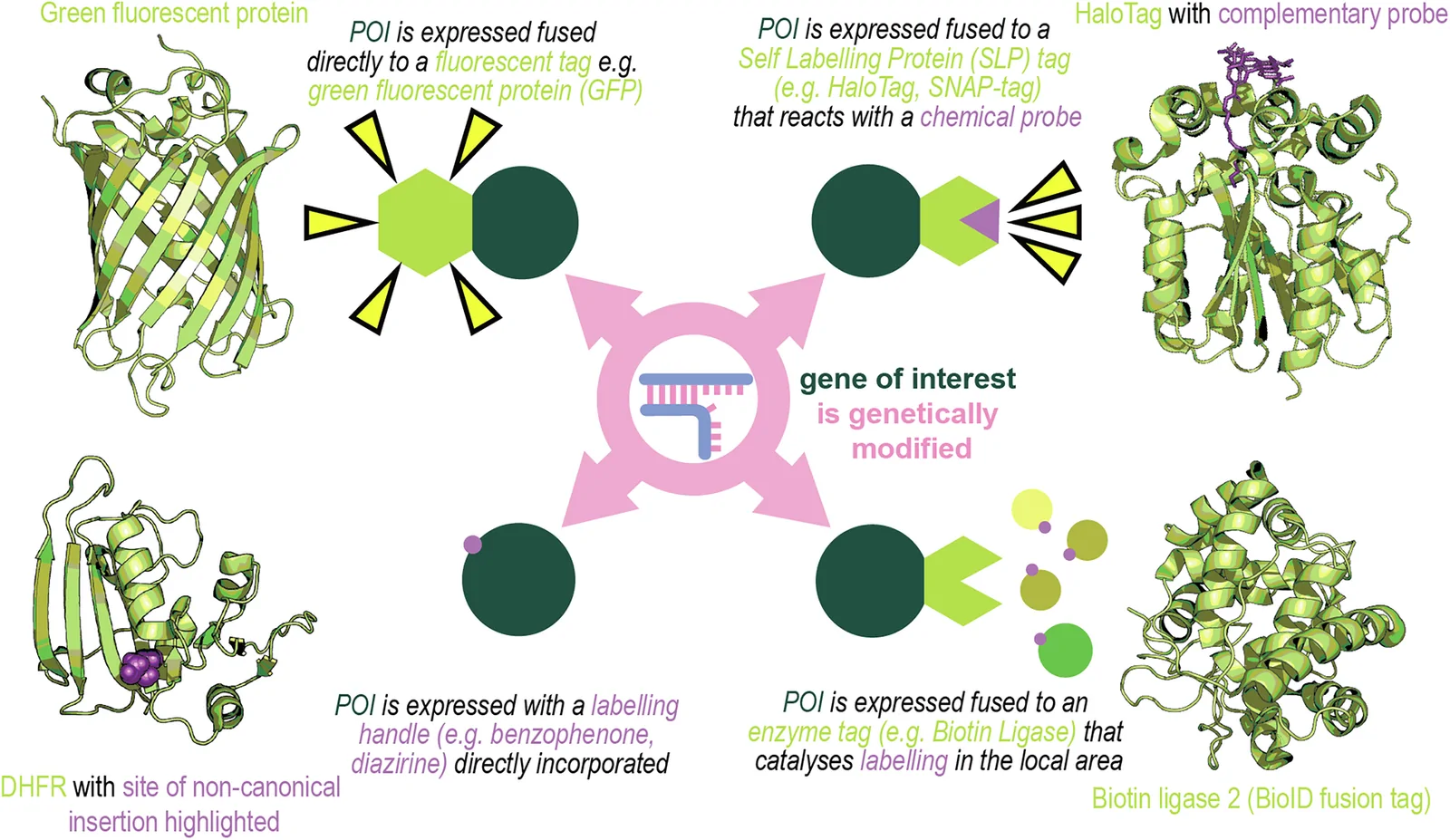
Examples of genetics approaches to protein labelling. Clockwise from top left: cartoon of a POI (forest green) fusion-tagged with GFP (limon) and structure of GFP (limon, PDB 1GFL). Cartoon of a POI (forest green) fusion-tagged with an SLP (limon) and structure of the HALO tag (limon, PDB 6U32) with the complementary probe highlighted (purple). Cartoon of a POI (forest green) fusion-tagged with a catalytic proximity-labelling group (limon) and structure of biotin ligase 2 (limon, PDB 1OAG), used in BioID. Cartoon of a POI (forest green) with a non-canonical amino acid inserted (purple) and structure of dihydrofolate reductase (DHFR, limon, PDB 6CXK), yellow triangles highlight the light-activated motif.

During the last 20 years, the development of genetic approaches to protein labelling (Fig. 1) has offered unrivalled specificity for labelling a protein of interest (POI). Perhaps most famously, Green Fluorescent Protein (GFP) fusion tagging emerged as an invaluable tool for imaging of live cells^7^. Subsequently, self-labelling protein tags (SLPs) such as SNAP-tag and HaloTAG were introduced. These small proteins can readily be generated as fusions with a POI, react with complementary small-molecule probes, and have emerged as important tools for labelling POIs with spatiotemporal resolution^8–10^. Proximity-based labelling^11^ has also facilitated biomolecular analyses: BioID, where a fusion protein incorporating a biotin ligase fused to the POI catalyses the biotinylation of nearby proteins, has been developed as a tool for identifying a POI’s interactome^12,13^. Meanwhile, genetic code expansion, where non-canonical amino acids loaded with reactive handles are inserted into a POI, has emerged as another versatile technique to enable a variety of powerful spatiotemporal interactome and localisation analyses^14,15^. New techniques continue to be developed; for instance, APEX and TREX methods are capable of labelling proteins and RNAs that interact with a POI or organelle, enabling further exploration of spatial relationships in and between cells^16,17^. All of the aforementioned methods have limitations: labelling requires engineered cells, genetic manipulation is often restricted to the *C*- or *N*-terminus, and fusion proteins add significant mass to the POI. The size of a labelling group is particularly important, as it can affect localisation, partner recognition, the dynamics of a protein and the kinetics of its function. Consequently, alongside the development of new analytical techniques, there is increasing interest in the development of chemical probes (bioactive ligands used to understand biology) for protein analyses in a *minimally perturbed* environment^18–20^.

Chemical probes^21,22^ – ligands that selectively interact with a POI – can allow temporary and titratable modulation of that POIs function, permitting evaluation of “druggability” and disease relevance; however, these can be of limited use for in-depth mechanistic analyses of cellular interactions of POIs. Complementary broad-spectrum approaches such as proteomics^23^, can yield powerful insight into features of the proteome such as the prevalence of post-translational modifications or expression of different proteoforms in response to a chemical probe. By leveraging covalent protein chemistry, more targeted analyses become possible as is described below.

### Covalent protein and ligand-directed chemistry

**Fig. 2:**
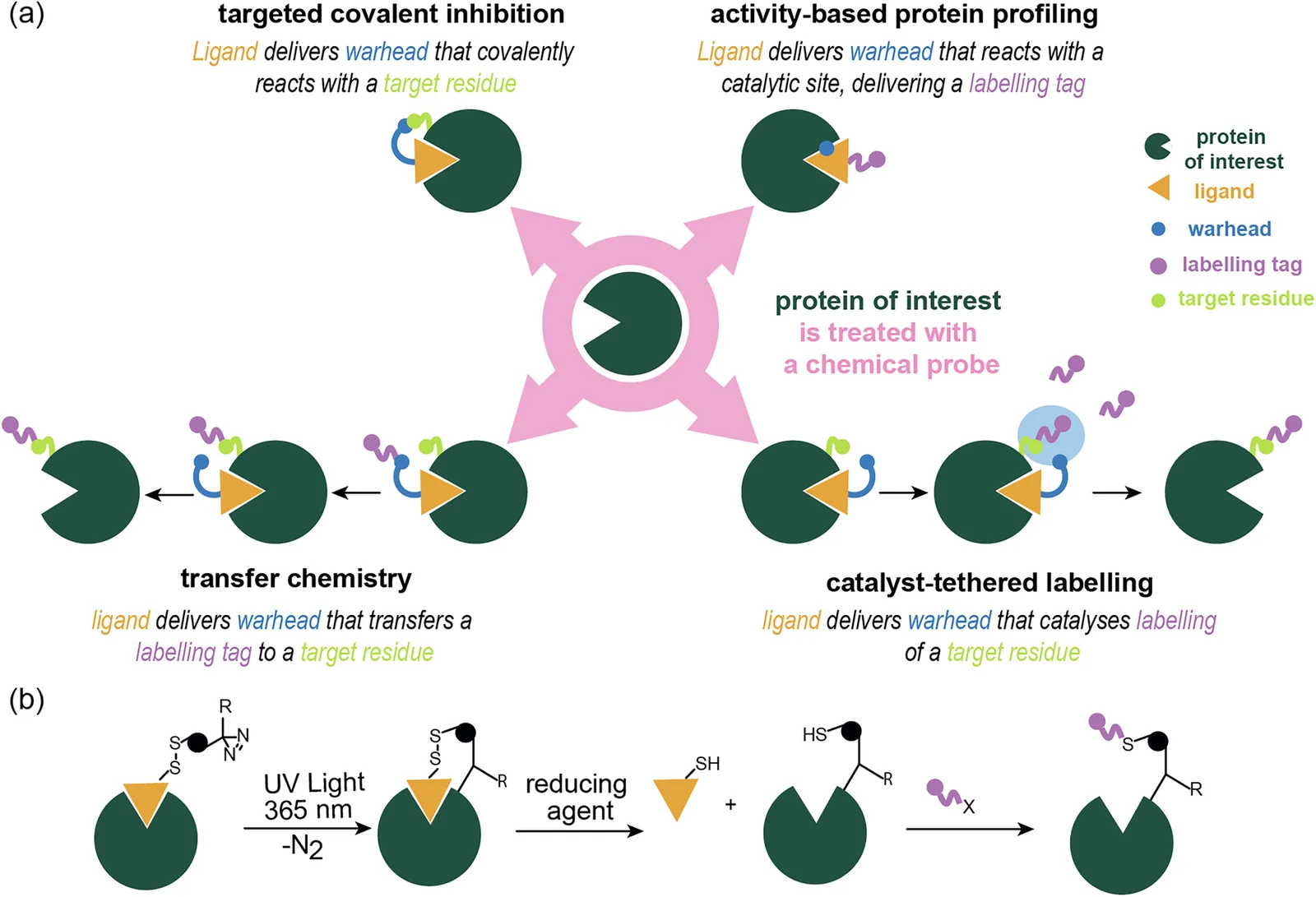
Examples of ligand-directed chemistry approaches. **a** Top: reaction between the ligand and the POI binding site results in inhibition of function at that site; bottom: a remote site can be labelled by transferring a group from the ligand to the protein or, by a catalytic group on the ligand that promotes POI labelling due to proximity effects; **b** schematic depicting a three-step t-LDL process whereby a diazirine attached to a ligand undergoes photoinduced crosslinking to a target protein. Disulphide reduction releases the ligand and subsequent labelling of the newly introduced thiol with an electrophile.

Covalent modulators (Fig. 2a) are receiving renewed interest for drug development and chemical biology studies, with a wide array of chemistries elaborated for this purpose^24–28^. Covalent modulators feature a reactive group (often termed the warhead), conjugated to a ligand that binds a POI; the warhead typically incorporates a weakly electrophilic reactive centre that can react with nucleophilic residues on the target protein. The reactive group should display minimal reactivity in the absence of the POI to maximise selective labelling. Commonly targeted amino acid side-chains for conjugation are cysteine and lysine, although chemistries for targeting other amino acid nucleophilic functionalities, including tyrosine, threonine and serine, have been established^24–28^. Covalent attachment of a modulator can lead to improved potency, selectivity, and duration of action; the reader is directed to reviews on covalent ligand discovery for more details^26–29^.

Ligand-directed labelling (LDL) chemistry is a related chemical biology approach where the ability of a ligand to selectively bind a target is exploited to drive a chemical reaction. Its power lies in its use for discovery biology, for instance in target validation and off-target identification^30–32^ using mass spectrometry-based proteomics, so-called chemical proteomics or chemoproteomics. The renaissance in the use of covalent modulators as drugs parallels the development of LDL. Reactive ligands have emerged as powerful tools to study biology; they exploit chemoselectivity to provide insight into the prevalence of reactive amino-acid side-chains in the proteome and can identify sites ripe for exploitation by covalent modulators^25,33,34^. However, without a recognition motif, such reactive ligands react indiscriminately with their target amino-acid side-chains throughout the proteome. In chemoproteomics techniques, such as activity-based protein profiling (ABPP), an inhibitor appended with a reactive group (warhead) and a reporter tag is used to covalently label a POI at the active site, giving quantitative readouts of enzyme activity^35^. However, labelling at the active site disrupts enzyme function, limiting the ability to study the roles of the POI in an unperturbed system. More recently, this labelling approach has been broadened to allow the exploration of ‘ligandable’ sites beyond the active site, such as a protein-protein interface, and the use of a greater diversity of binding motifs, including fragments^36,37^. However, reagents that result in selective protein modification whilst maintaining native POI function are rare; instead, POI function is typically abrogated.

### ‘Traceless’ ligand-directed labelling chemistry

‘Traceless’ ligand-directed labelling (t-LDL) seeks to label a POI with minimal disruption to its structure, function and localisation, making t-LDL a powerful technology for studying proteins in their endogenous environment. t-LDL relies on proximity-assisted rate enhancement to achieve labelling with surgical precision (Fig. 2a). The combination of the ligand driving selectivity for the target protein, a linker to provide access to more distal residues, and the chemoselectivity of the warhead allow the design of probes that label a specific target residue away from a protein’s active or canonical binding site. In this regard, determinants of t-LDL reagent effectiveness (labelling rate and second-order rate constant) parallel those of covalent inhibitors. Moreover, by transferring only a small labelling group to an adjacent, non-functional site, t-LDL offers the advantage of minimising the impact of labelling on the biophysical and biochemical behaviour of the POI^19,38^. t-LDL chemistries can be broadly divided into two categories, namely transfer t-LDL and catalyst-tethered t-LDL (Fig. 2). In transfer t-LDL, the warhead itself cleaves during reaction with the target residue, transferring the labelling tag to the POI while allowing the ligand to dissociate. In catalyst-tethered t-LDL, the warhead is a catalytic group that drives the reaction between a residue on the POI and a separate probe or tag.

### Transfer t-LDL

t-LDL was exemplified early on by Hamachi and co-workers (Fig. 2b) in efforts to develop biosensors for lectins by using a sugar linked through a readily cleavable disulphide to a diazirine^39,40^. Following proximity-driven photoinduced crosslinking, cleavage of the disulphide allows removal of the sugar (the ligand) and consequent liberation of the binding site; the newly introduced thiol on the POI can be conjugated to a fluorophore. This two-step method was adapted by Horne et al. for structural proteomics^41^. Whilst the stability of the disulphide in a cellular environment and the requirement of a reduction step might be considered problematic, the approach was elaborated by Li and co-workers to enable analyses of binding sites for post-translational modification (PTM)-dependent epigenetic protein-protein interactions^42^. The use of crosslinking chemistry alongside disulphide exchange has thus been elaborated to facilitate powerful interactome analyses. Nevertheless, the strategy has limitations in a t-LDL context, namely the largely indiscriminate crosslinking behaviour of diazirines^41,43^ and the requirement for chemical modification of the crosslinked protein using reducing agents. The underlying concept, however, showed promise and provided motivation to develop single-step, site, and therefore amino-acid selective transfer chemistries, to enable precision labelling.

### Cysteine-targeting warheads for transfer t-LDL

**Fig. 3:**
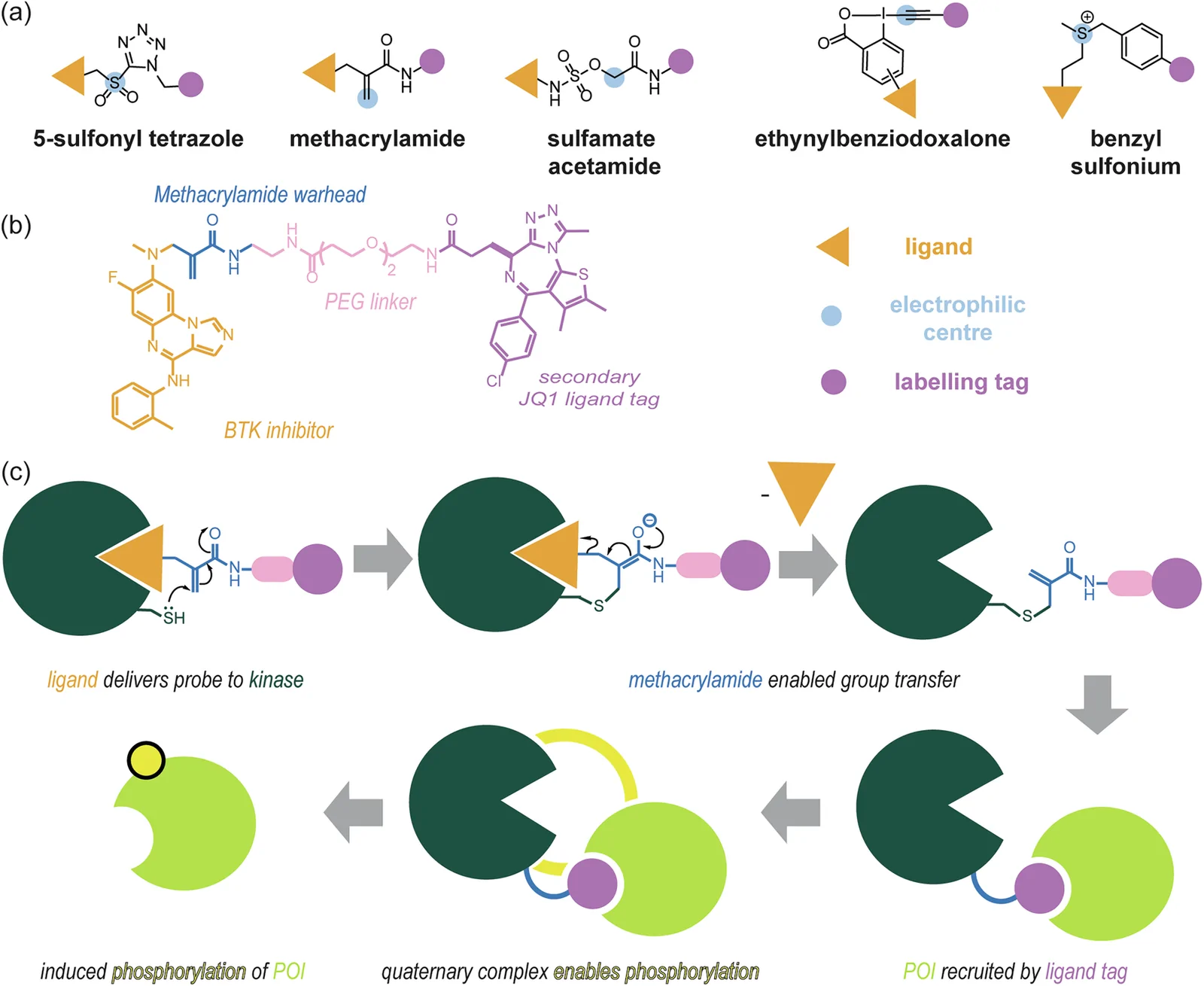
Cysteine-targeting t-LDL chemistry. **a** Structures of cysteine-targeting t-LDL warheads; **b** Structure of the bifunctional LDL probe designed by Pergu et al.; **c** Schematic illustration of how PHICS has been used to phosphorylate a target POI. Kinase in forest green, POI in limon and phosphorylation represented in yellow.

While cysteine-targeting warheads are the most studied in the context of covalent inhibitors^44^, there has been less development of cysteine-targeting t-LDL probes. One reason for this is that many of the classical cysteine-targeting warheads, such as acrylamides, epoxides and aryl halides, whilst productive in the context of covalent inhibitors, are unsuitable for t-LDL chemistry. Nonetheless, examples of LDL-compatible cysteine-targeting warheads do exist (Fig. 3a). An early example thought to target cysteine employed a 5-sulfonyl tetrazole probe which, when linked to cyclosporin A, was used to tag its target, cyclophilin A, by nucleophilic aromatic substitution^45^.

The London group recently reported the successful adaptation of acrylamide warheads for covalent ligand-directed release (CoLDR) chemistry^46^. Appending a methacrylamide warhead with an α-substituted heteroatom resulted in a versatile cysteine-targeting transfer-warhead. Using this warhead, several ‘turn-on fluorescence’ probes were designed, where the fluorescence of the cleaved leaving group could be used as a readout for successful binding in high-throughput screening. Subsequently, site-selective t-LDL probes were developed for a range of target proteins, including Bruton’s tyrosine kinase (BTK), K-Ras and the SARS-CoV-2 papain-like protease. Probes using α-substituted methacrylamide warheads were used to label BTK in cells; that labelling did not compromise enzyme activity validated this warhead as a useful tool. This CoLDR warhead strategy has also been used to design several potent PROTACs for BTK^46,47^.

Sulphamate acetamides, also developed by the London group, represent another adaptation of a classical cysteine-targeting warhead into one suitable for transferase t-LDL. Replacing the chloro substituent in an α-chloroacetamide warhead with a sulphamate yielded a cleavable, efficient and biocompatible electrophile that was successfully employed for CoLDR chemistry in vitro and *in cellulo*^48^. CoLDR chemistry has recently been used as a group-transfer warhead to create diverse, small molecule-functionalised fusion constructs with POIs to facilitate analysis of biomolecular mechanisms^49^.

Further recent methods for cysteine targeting include the use of peptides appended with hypervalent iodine reagents, which allow site-directed ethynylation of cysteine residues or formation of vinylbenziodoxolone (VBX) adducts on the KELCH domain of KEAP1^50^. Similarly, sulfonium probes have been used to label a specific cysteine in a model PDZ protein (PDZ-RGS3)^51^.

A particularly notable application of cysteine-targeting t-LDL is the work of Pergu et al. on phosphorylation-inducing chimeric small molecules (PHICS)^52^. By appending a known kinase inhibitor with a methacrylamide warhead and a secondary protein ligand, JQ1, the authors produced bifunctional small molecules capable of tagging the target kinase with JQ1, allowing for recruitment of BET family proteins, e.g., BRD4, to the kinase (Fig. 3b); this allowed for induced phosphorylation of a POI and represents a significant step forward in the tools available for studying protein phosphorylation, a critical PTM implicated in many disease pathways and cell-signalling processes. The authors designed PHICS that recruited an epigenetic factor (BRD4) implicated in cancer to a kinase (BTK) to induce its phosphorylation (Fig. 3c). Since phosphorylation is critical to cell signalling, PHICS also represent a way to induce signalling cascades, and have allowed the authors to induce phosphorylation of downstream targets and even cell death in ibrutinib-resistant cancer cells^52^.

### Lysine-targeting warheads for transfer t-LDL

**Fig. 4:**
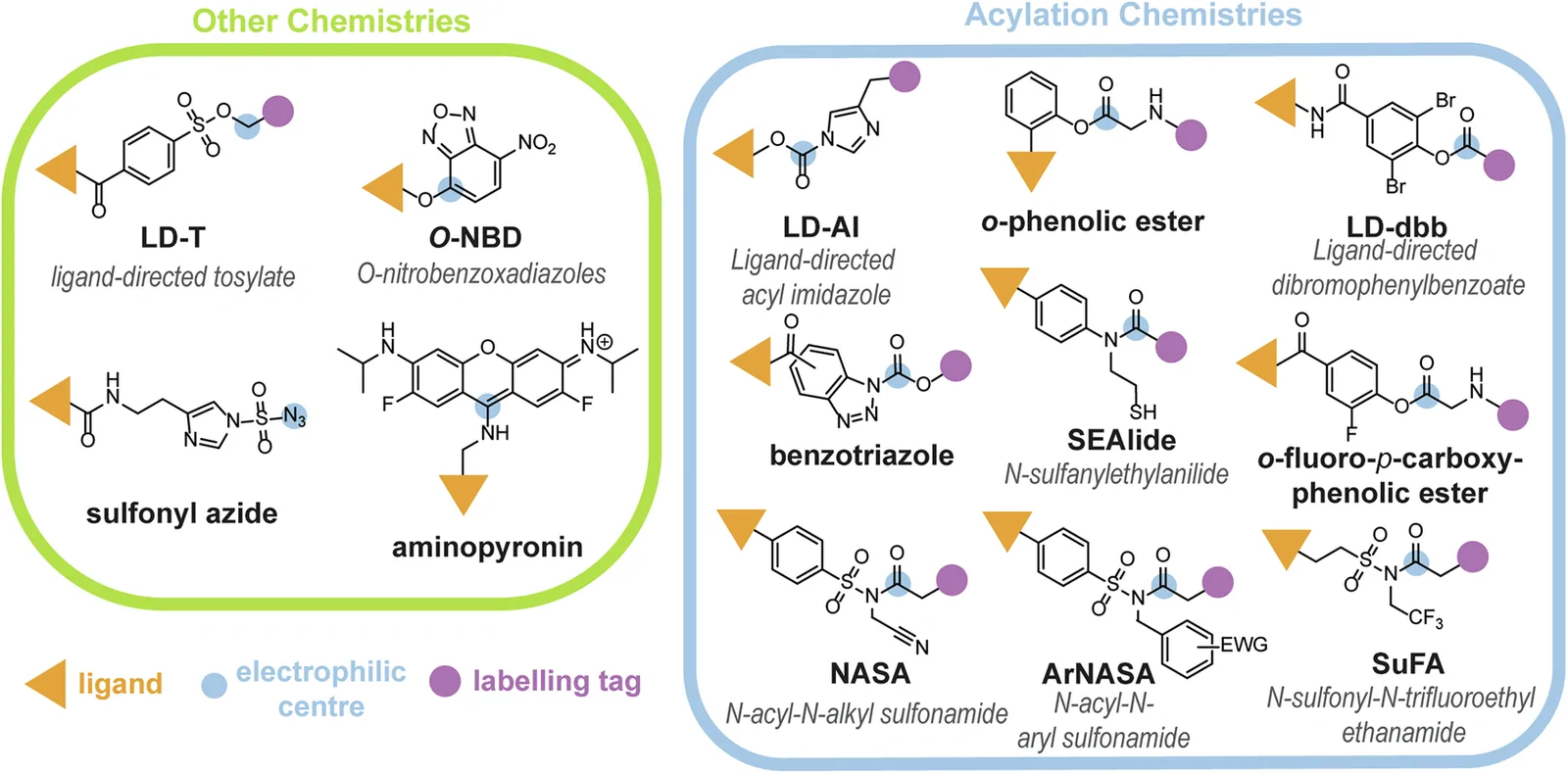
Lysine-targeting t-LDL chemistries. Generalised structures of t-LDL reagents highlighting the ligand for a POI, the tag or labelling group and the reactive centre.

While there are fewer reported chemistries for labelling lysine than cysteine, there has been greater development of lysine-targeting t-LDL probes; this can be attributed to the much greater abundance of lysine (5.9%) than cysteine (2.3%) on protein surfaces, making it a more attractive target^53^, and the pioneering work of the Hamachi group^54^. As a result, a wide range of chemistries are available for lysine-targeting t-LDL, with examples of alkylation, nucleophilic aromatic substitution (S_N_Ar) and diazo transfer, although the majority use acylation chemistry (Fig. 4).

The Hamachi group has been developing LD chemistries for two decades^55,56^, mostly to target lysine residues. Notable early examples include the ligand-directed tosyl (LD-T)^55^, acyl imidazole (LD-AI)^57^ and dibromophenyl benzoate (LD-dbb)^58^ warheads; these have been used to design probes for a wide range of protein targets to carry out labelling in vitro, *in cellulo* and in vivo^55–60^.

**Fig. 5:**
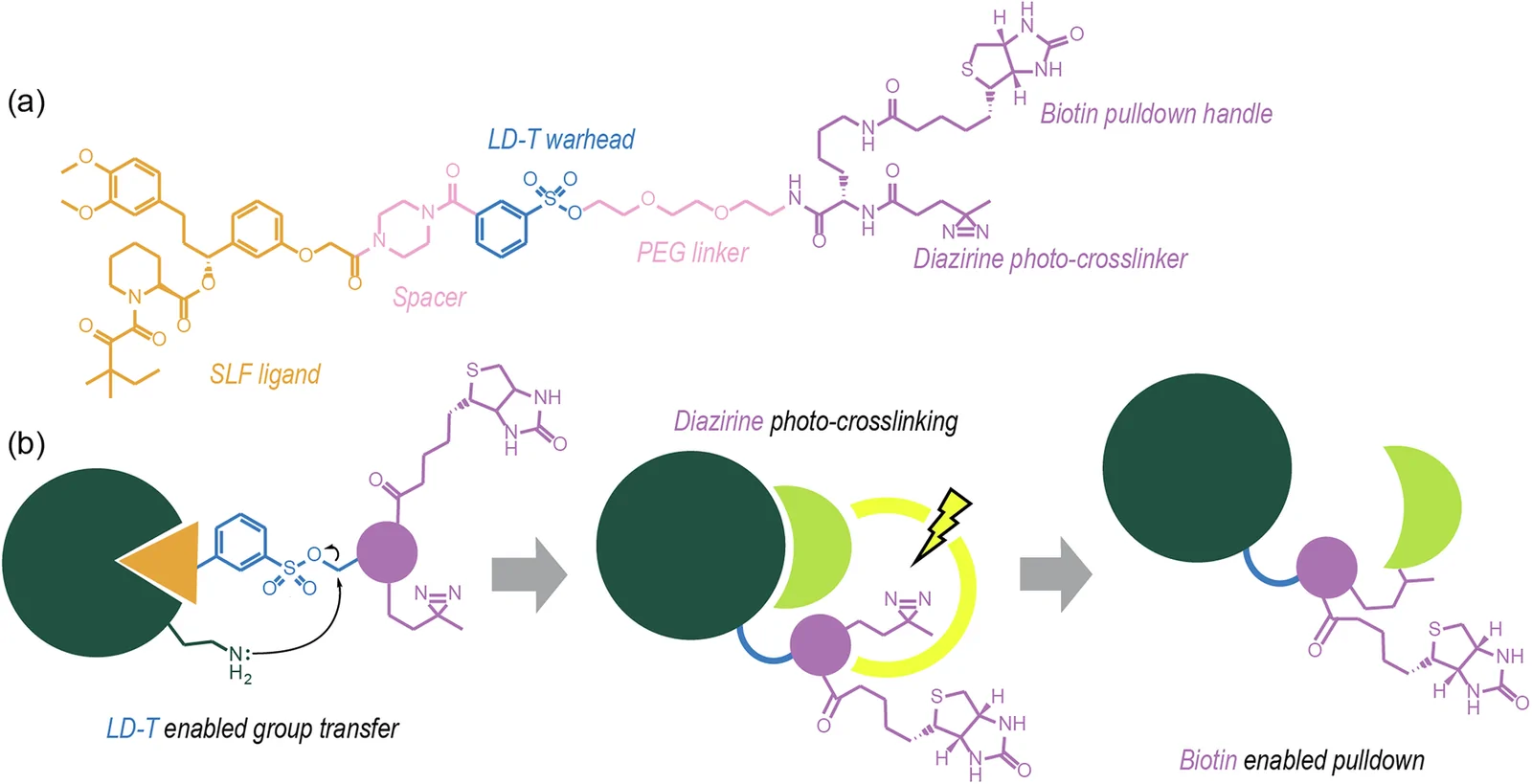
LD-T t-LDL. **a** Structure of bifunctional LD-T probe utilised by Tamura et al.; **b** Schematic illustration of PPI identification using this LD-T probe.

**Alkylation:** Ligand-directed tosyl (LD-T) chemistry has emerged from the earlier warheads as the most widespread, largely thanks to its promiscuous activity. Despite being developed to target lysine residues, LD-T warheads have also been reported to label His, Glu, Asp, Tyr and Cys residues^38^. The work of Tamura et al. using a variety of LD-T probes to investigate FKBP12 is a notable early application of LDL^59^. Although LDL chemistry was still in its infancy, with the probes sometimes requiring days to label the protein in yields of ~10–45% (labelling rate (*k*_*L*_) ~ 6 × 10^−6^ and second-order rate constant (*k*_*L*_/*K*_*d*_) ~ 4 × 10^1 ^M^−1^s^−1^)^61^, this work stands as an impressive proof of concept by moving LDL towards living systems analyses and performing complex LDL experiments. One probe, created by appending a synthetic FKBP12 ligand with an LD-T warhead attached to a bifunctional tag containing a biotin handle and a diazirine, could pull down FKBP12 binding partners *in cellulo* (Fig. 5). This experiment represents the first reported example of utilising LDL probes to track PPIs in cells^59^.

**Acylation:** Simple esters might be expected to serve as ideal lysine-reactive warheads for t-LDL chemistry; however, they also pose concerns over hydrolysis and/or reactivity towards other nucleophiles. Despite these potential issues, several ester-based t-LDL reagents have been developed. An early example employed marinopyrrole functionalised with an *ortho*-phenolic ester to acylate a single lysine residue (Lys115) on the identified target (actin) in cells^62^. In a more recent example geared towards PTM rewiring, KHAc, a derivative of a phosphoglycerate mutase 1 (PGAM1) inhibitor, was shown to transfer its acetyl group to the ε-NH_2_ of Lys100, resulting in suppression of PGAM1 enzymatic activity^63^. A series of GPCR labelling reagents that employ a *para*-carboxy-*ortho*-fluorophenolic ester have also been reported by the Hill and Kellam groups^64–66^, while Rosen et al. introduced the use of duplex nucleic acids for labelling using a metal-chelating motif on one strand and an *N*-hydroxysuccinimide acylating group on the other strand. Following reaction with suitable nucleophiles on the protein target, non-covalent strand displacement unveiled the binding site^67^. This approach has been used with other binding modalities^68,69^.

Denda et al. introduced the *N*-sulfanylethylanilide (SEAlide) as a reactive unit to facilitate acylation. The reaction relies on *N* > *S* acyl transfer as observed in inteins. The SEAlide is unreactive in the absence of the protein target, even to a large excess of amine or cysteine methyl esters, whereas in the presence of the protein (either carbonic anhydrase or cyclooxygenase 1) the *N-S* acyl transfer and subsequent nucleophilic exchange on the thioester group are observed. The authors hypothesise that this is due to proximal acidic and basic groups on the protein surface activating the SEALide reagent; however, they did not report direct evidence of this, which highlights both the importance of the local protein environment to t-LDL chemistries and the inherent difficulty of characterising such effects^70^. Acylation has also been used to label antibodies by using ligands for the FC domain of the heavy chain of IgG^71,72^; this has allowed the preparation of antibody-drug conjugates in vivo to prolong the lifetime of the active drug^72^.

Ligand-directed (alkoxyl)acyl imidazole (LD-AI) chemistry is a related chemistry that has proven useful for labelling cell-surface receptors^57^, including receptors at the synaptic junction in living mouse brains^73^. Upon reaction with lysine, these types of t-LDL reagents lead to carbamate linkages between the tag and protein (*k*_*L*_ ~ 6 ×10^−6^ s^−1^ and *k*_*L*_/*K*_*d*_ ~ 1 ×10^1^M^−1^s^−1^)^61^. Benzotriazoles in place of imidazole were recently developed and employed by the Wu group^74^, who showed these reagents rapidly generate carbamates upon reaction with lysine and subsequent loss of the POI binding motif as the leaving group.

A more recent example of a lysine-targeting t-LDL warhead, and perhaps the most widely adopted to date, is the *N*-acyl-*N*-alkyl/aryl sulphonamide (NASA/ArNASA) warhead, developed by the Hamachi group^61,75,76^. Initially developed as the *N*-alkyl (NASA) variant, the warhead showed good selectivity for lysine residues (with some reactivity towards tyrosine also reported), favourable kinetics (*k*_*L*_ ~ 1 ×10^−2^ - 8 × 10^−3^ s^-1^ and *k*_*L*_/*K*_*d*_ ~ 7 ×10^1^ - 3 × 10^4^ M^−1^s^−1^ comparable to the faster bioorthogonal reactions of e.g. tetrazine ligations)^61^ and a faster labelling rate compared to other lysine-targeting chemistries (e.g. LD-AI: *k*_*L*_ ~ 6 × 10^−6^ s^−1^ and *k*_*L*_/*K*_*d*_ ~ 1 × 10^1^ M^−1^s^−1^ or LD-dbb: *k*_*L*_ ~ 3 × 10^−4^ s^−1^ and *k*_*L*_/*K*_*d*_ ~ 6 × 10^2^ M^−1^s^−1^)^61^. This warhead has since been successfully used in conjunction with a variety of different ligands, including small molecules^77,78^, metal complexes^79^ and oligonucleotides^80,81^, to label protein targets in vitro and *in cellulo*. Concerns over the NASA warhead’s intracellular stability and off-target reactivity with cysteine and tyrosine residues led to the development of second-generation *N*-aryl (ArNASA) probes^76^, whereby substituting the alkyl group for an electron-deficient aryl group improved the warhead’s tuneability, hydrolytic stability and specificity towards lysine residues (*k*_*inact*_ =, 1.8 × 10^–3^ s^–1^ vs 3.5 × 10^–3^ s^–1^ and *k*_*inact*_/*K*_*d*_ = 2.9 × 10^4^ vs 8.5 × 10^4^M^–1^ s^–1^ for a NASA-functionalised and ArNASA-functionalised HSP90 covalent inhibitor respectively). Although initially reported as a warhead for covalent inhibitors, the ArNASA warhead is also capable of transfer t-LDL.

NASA/ArNASA-based LDL probes have been used to study a variety of proteins within their native contexts, including the fluorescent labelling of GPCRs (CB2R and A2BAR)^82^ and kinases (PTK7)^80^ in live cells, and as a tool for activity-based profiling of matrix metalloprotease MMP12^83^. The warhead has also been used for the development of covalent inhibitors by the Hamachi group and others^34,61,77^, development of photo-controlled enzymes^84^, as a probe for high-throughput in-cell compound screening^78^, as a chemoproteomic tool to enable direct mass spectrometric detection of active proteases by Matrix-Assisted Laser Desorption/Ionisation (MALDI)^85^ and as a warhead in a targeted protein degradation strategy^86–88^.

Deiters and co-workers have developed several probes that utilise the NASA warhead in conjunction with aptamers^80,81^. Aptamers are short, single-stranded nucleic acid molecules with well-defined secondary structures, capable of binding a target protein with high affinity and specificity. The Deiters group has exploited this, in combination with the NASA warhead to image challenging targets. Tivon et al. demonstrated that incorporation of an alkyne-containing nucleotide into the aptamer during synthesis enabled facile modular synthesis of NASA–aptamer conjugates^81^, and in subsequent work by Albright et al., NASA–aptamer probes were used for cell-surface imaging of PTK7^80^.

**Fig. 6:**
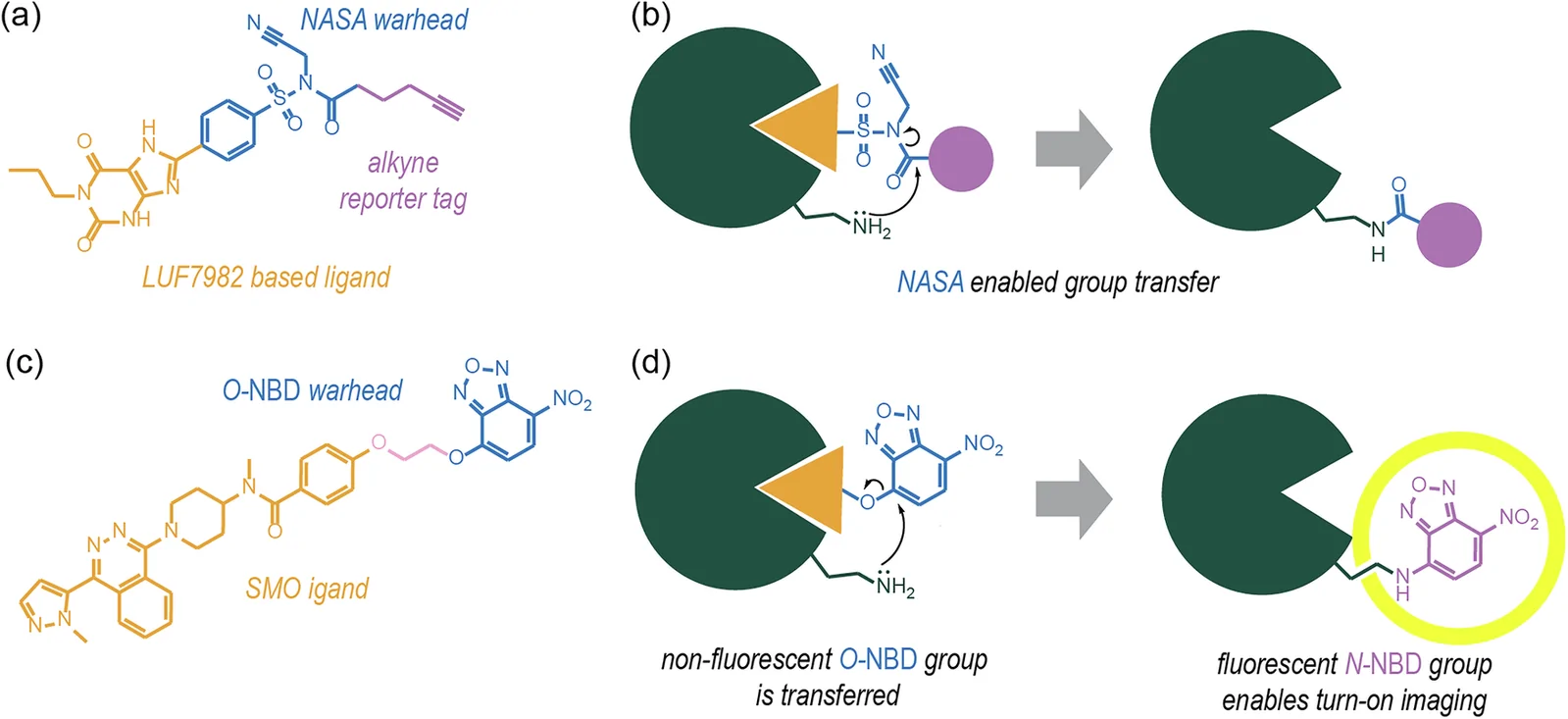
Lysine-targeting t-LDL. **a** Structure of NASA probe utilised by Beerkens et al.; **b** Schematic illustration of NASA LDL; **c** Structure of *O*-NBD probe utilised by Xue et al.; **d** Schematic illustration of *O*-NBD ‘turn-on’ fluorescence.

Beerkens et al. reported another application of the NASA warhead, specifically the labelling of G protein-coupled receptors (GPCRs)^82^. GPCRs are a family of membrane proteins that are ubiquitous in many physiological processes; they have been the target of numerous drug discovery programmes, which has led to a relative abundance of known ligands and small-molecule probes for this important class of protein. However, most of these compounds bind at orthosteric sites, limiting researchers’ ability to study active GPCRs and how these proteins respond to ligand-induced activation. LDL is well positioned to overcome this problem since the ligand dissociates after transferring the labelling group. Beerkens et al. sought to exploit this feature by appending a known GPCR antagonist with a NASA warhead equipped with an alkyne handle (Fig. 6a, b). While the resulting probes were capable of labelling in a manner that retained some agonist sensitivity and were suitable for live-cell labelling, Beerkens et al. noted issues with the reporter tag inhibiting some ligand-induced activation, as well as significant non-specific labelling, attributed to steric hindrance of ligand binding and the NASA warhead’s high intrinsic reactivity, respectively^82^. Notably, in the intervening period, Hamachi and co-workers reported that second-generation ArNASA warheads have lower intrinsic reactivity and are more tuneable, overcoming the specificity issues of the first-generation warheads used by Beerkens et al.^76^.

A more recent addition to the NASA portfolio of reagents is the *N*-sulfonyl-*N*-(trifluoroethyl)ethanamide (SuFA) warhead, described by Singh et al.^89^. In studies geared towards development of group-transfer chimeras (GRCs), where transfer of a conventional NASA warhead to a lysine proximal to the ligand binding site was desired, labelling at both proximal and distal lysine residues was observed for multiple kinases. The use of the SuFA warhead attenuated reactivity, including hydrolytic instability, and facilitated more selective labelling of the target lysine residues (observed rate constant (*k*_obs_) for SuFA probe labelling of VEGFR2 1.1 × 10^−3^ s^−1^ vs NASA probe 3.4 × 10^−3^ s^−1^).

The diverse and successful applications of NASA-based LDL chemistry in a variety of contexts, and the ability to target lysine, a much more abundant but relatively under-targeted residue compared to cysteine, render this warhead a powerful chemical biology tool. Additionally, the development of more tuneable *N*-acyl-sulphonamide-based groups to mitigate some of this warhead’s drawbacks can be expected to lead to more widespread exploration of its potential.

**Other Lysine-Reactive Chemistries:** S_N_Ar chemistry has also been used to label lysine residues. For example, moderately electrophilic aminopyronin fluorophores were selectively transferred between lysine residues at protein–protein interfaces using aminopyronin-conjugated immunoglobulin G (IgG) to label Protein A (SpA)^90^. *O*-Nitrobenzoxadiazoles (*O*-NBD) were utilised by Gober et al. for affinity labelling of histones^91^, by Yamaguchi et al. to design LDL-based turn-on fluorescence probes^92^, and by Xue et al. for fluorescence labelling of the smoothened receptor (SMO) (Fig. 6c, d)^93^. Aldehydes have also been used for labelling: reductive amination of lysine residues has been used in two-step protocols that employ the aforementioned strand displacement strategy or a cleavable linker between a POI ligand and reactive warhead^94,95^. Finally, site-specific diazo transfer to lysine has been achieved using BioY/streptavidin and a biotin-functionalised imidazole-1-sulfonyl azide group^96^.

### Tyrosine-targeting chemistry for transfer t-LDL

**Fig. 7:**
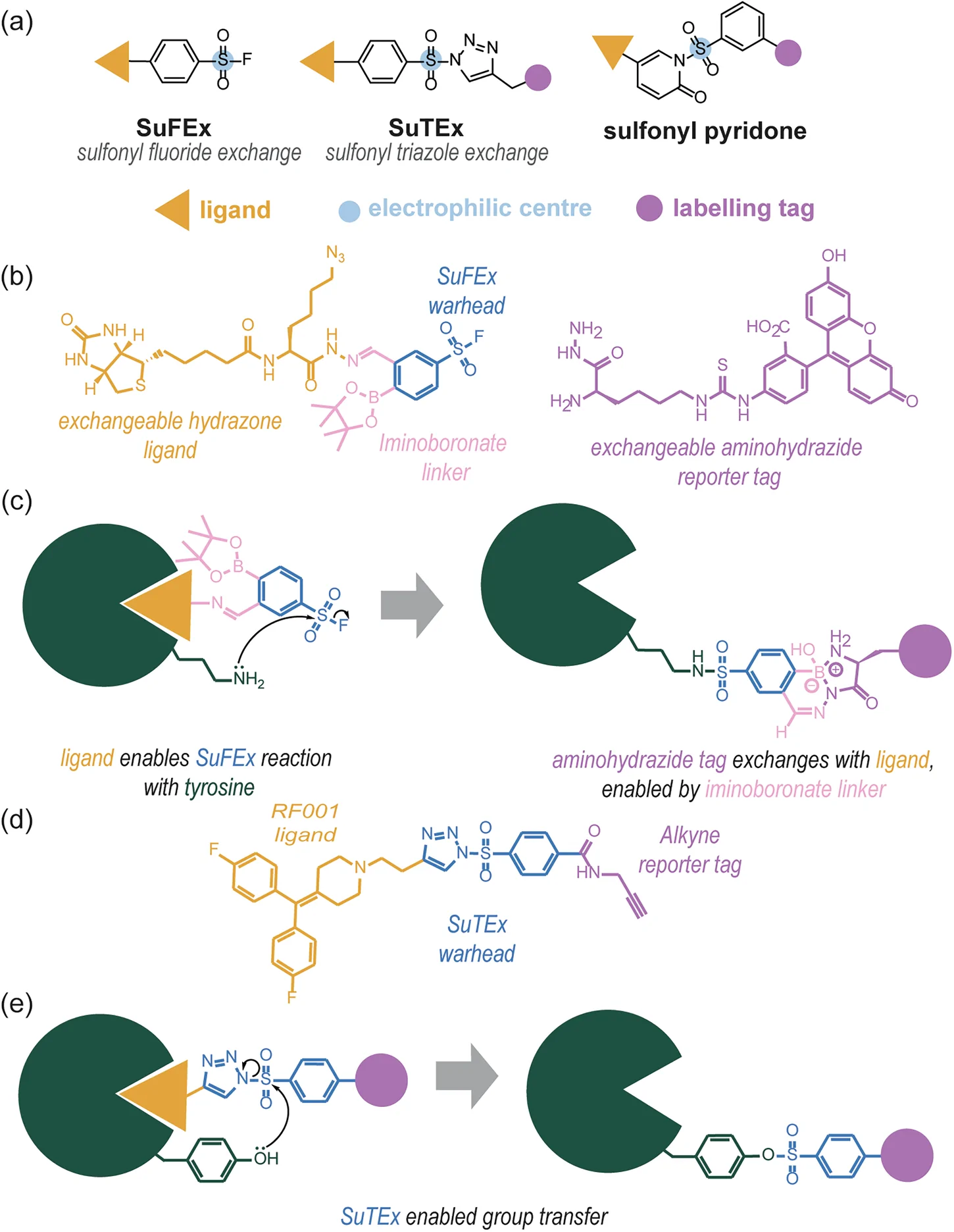
Tyrosine-targeting t-LDL. **a** Structures of tyrosine-selective t-LDL warheads; **b** Structure of the SuFEx iminoboronate probe and exchangeable tag utilised by van der Zouwen et al.; **c** Schematic illustration of SuFEx iminoboronate t-LDL; **d** Structure of the SuTEx probe utilised by Huang et al.; **e** Schematic illustration of SuTEx t-LDL.

Due to the aromaticity of its phenol side-chain, tyrosine is less nucleophilic than cysteine or lysine. This makes tyrosine-targeting warheads more difficult to develop, as a probe must balance sufficient reactivity and stability in biological contexts, while displaying chemoselectivity for a phenol, factors that are often at odds. Nonetheless, several chemistries for targeting tyrosine have been reported, notably the sulfonyl-fluoride exchange (SuFEx) reaction that was brought back to prominence by Dong et al. in 2014 (Fig. 7a)^97^. The related sulphur-triazole exchange (SuTEx) warhead is notable as a transfer t-LDL-compatible chemistry that primarily targets tyrosine (with some reactivity towards lysine)^98^. While similar to the more established (and also Tyr/Cys/Lys reactive) SuFEx chemistry^97,99^, changing the leaving group in the warhead to a triazole enhances reactivity and chemoselectivity towards tyrosine and provides a more tuneable leaving group^100^. Indeed, as the warhead was being developed, it was found that adding a phenyl substituent to the triazole leaving group enhanced reactivity and chemoselectivity; these effects could be further tuned via *para*-substitution of the phenyl group^100^.

Adaptation of SuFEx and SuTEx chemistry for t-LDL is less explored. For SuTEx and SuFEx warheads, the sulphur acts as the site for nucleophilic attack and remains bound to the target residue^20,99,101^; thus, to design a traceless LDL probe it is necessary for the ligand to be appended to the leaving group, which is problematic for SuFEx where the leaving group is fluoride. One example where a SuFEx group has been used for t-LDL involved the use of an aptamer bearing a SuFEx group to tag thrombin: following covalent labelling, addition of a complementary strand for the aptamer, resulted in duplex assembly and liberation of the aptamer binding site^102^. A related two-step method based on reversible covalent chemistry of iminoboronates was introduced by the group of Witte, whereby the affinity ligand was connected to a sulfonyl fluoride warhead. Following labelling of the target protein, the iminoboronate could be cleaved by a suitable amine nucleophile (Fig. 7b, c)^103,104^. This approach was applied to carbonic anhydrase 2 and BioY and is a notable example of how proximity and tuneable chemoselectivity can be leveraged in t-LDL, as the SuFEx warhead actually labelled a lysine residue in this study.

To date, SuTEx warheads have been employed for chemical proteomics studies, leveraging the warhead’s tyrosine selectivity. Global proteome-scale studies utilised a SuTEx-based system to identify and profile hyper-reactive tyrosines^98^, and to develop a chemical phosphoproteomics approach for investigating post-translational modifications of tyrosine in live cells^105^. SuTEx-based LDL probes have been reported, including a general kinase-selective probe capable of traceless labelling in live cells and lysate (Fig. 7b, d)^105^. Beyond this, SuTEx warheads have been used in fragment-based ligand discovery and phenotypic screens^101^, and to develop several covalent inhibitors^100,101^.

The *N*-sulfonyl pyridone motif has also been used to develop reagents capable of cell-surface and intracellular labelling of a range of proteins including carbonic anhydrase 2 and 12, folate receptor, intracellular heat shock protein (HSP) 90 and lipopolysaccharide-induced cyclooxygenase 2^106^. Like SuFEx and SuTEx warheads, the *N*-sulfonyl pyridone group exhibits a preference for labelling of tyrosine but with some labelling of lysine. In a rare example where different transfer t-LDL chemistries were explored in parallel, *O*-NBD, *N*-sulfonyl pyridone and NASA warheads were used in t-LDL on the cannabinoid receptor type 2. Whilst the *O*-NBD reagent proved effective for exploring the delivery of a fluorophore, the ability to deliver a range of different functionalities motivated exploration of the latter two warheads, with the NASA-derived ligand proving more effective in labelling experiments^107^.

### Chemistry for t-LDL on other amino acids

**Fig. 8:**
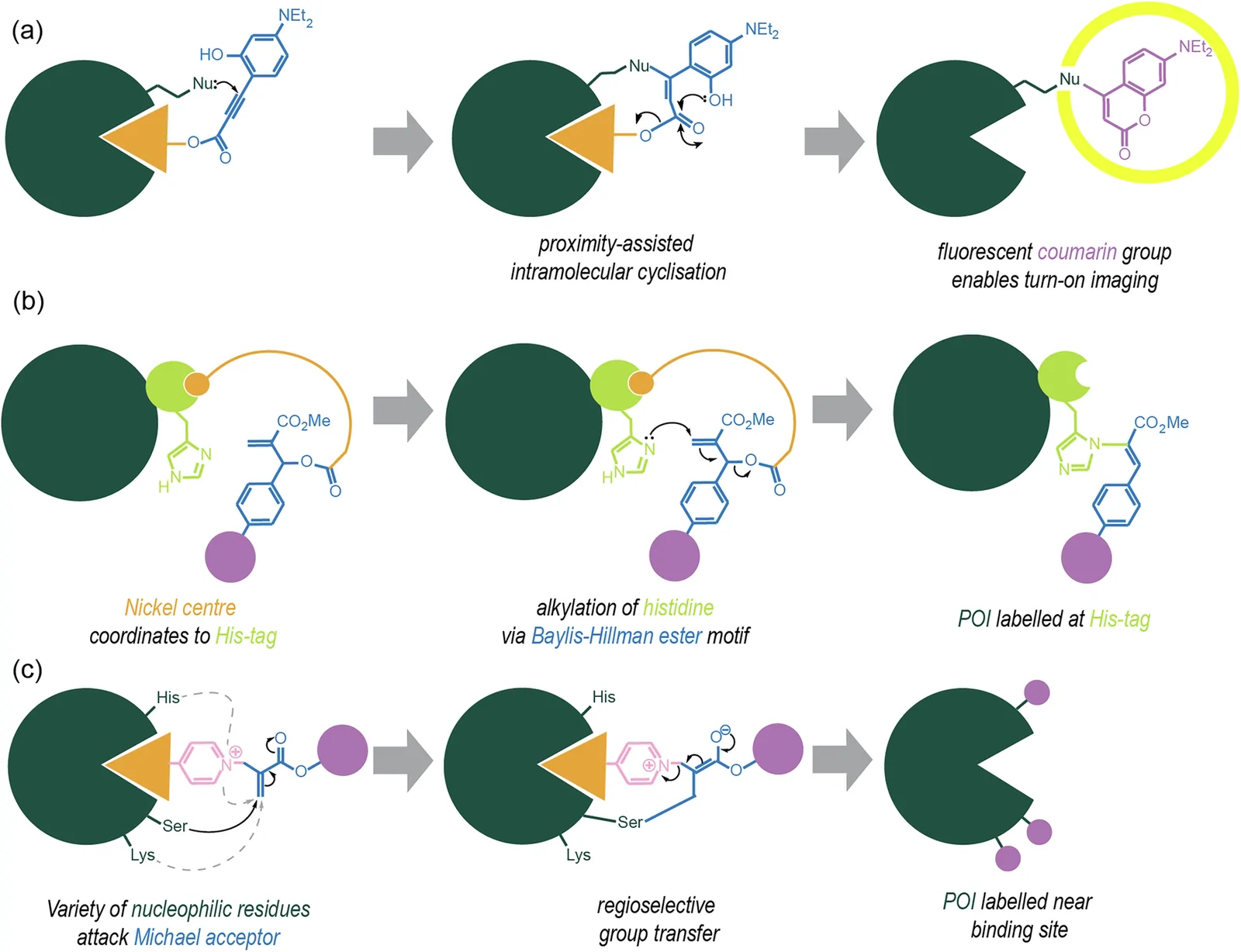
t-LDL for other residues. **a** Schematic illustration of turn-on fluorescence *via* ynoate probes developed by Kojima et al.; **b** Schematic illustration of histidine alkylation via Baylis-Hillman ester probes by Liu and Melman; **c** Schematic illustration of t-LDL-enabled binding-site mapping by Kurbanov et al.

Kojima et al. described a t-LDL reagent which was shown to label a range of nucleophilic amino acids^108^. This elegant work employs an enoate, generates an enoate upon reaction with a nucleophilic amino acid, subsequent formation of a coumarin, and turn-on fluorescence (Fig. 8a). The method was applied to small-molecule ligands for vitamin D receptor (VDR) where histidine was labelled, and to the co-activator binding site on VDR and peroxisome proliferator-activated receptor γ (PPARγ), where the authors proposed a lysine as the site of labelling. Liu and Melman reported the use of Baylis-Hillman esters conjugated to nickel-binding nitrolotriacetate for binding a hexahistidine tag and alkylation of imidazole (Fig. 8b)^109^. Finally, triggerable Michael acceptors bearing a pyridinium leaving group have been used to label proteins at multiple sites to map a binding site by reacting with histidine, lysine, and serine nucleophiles (Fig. 8c)^110^.

### Summary of transfer t-LDL chemistries

The key strength of transfer t-LDL is the ability to target specific residues *via* tuned warhead chemoselectivity that can be accessed. This is exemplified by some impressive applications of transfer t-LDL chemistries that have been used to study and even modulate complex biological systems, such as Pergu et al.’s adaptation of the methacrylamide warhead to produce PHICS and the wide variety of applications of the NASA warhead family^52,61,77–80^. However, transfer t-LDL is not without its challenges. The work of Beerkens et al. and Kosar et al. demonstrates that complications can arise from warhead stability, specificity, and mode of action^82,107^. Amongst the different parameters, reactivity of the warheads (e.g., electrophilic warheads will have some reactivity towards most nucleophilic side chains) and availability of a suitable proximal amino-acid side-chain, affinity of the ligand for the target protein, quantitative on/off target reactivity, local environment (incl. pH), rate of labelling in biological contexts, nature/effect of any linker between protein-ligand and reactive group, effect of labelling on protein structure and function all play a role. Some of the complicating factors here are fundamental; thus, transfer t-LDL chemistry relies on proximity-assisted rate enhancement and thus is sensitive to changes in local protein environment, which makes it difficult to predict which warhead (or warhead variant) to use based on warhead intrinsic reactivity studies. It should be noted that recent studies have systematically explored the potential for electrophile activation – referred to as latency – for covalent modulators: intermolecular labelling rates (*k*_*inter*_ (acylimidazole) ~ 10^−3^ s^−1^, *k*_*inter*_ (NASA) ~ 10^−1^ s^−1^ and *k*_*inter*_ (SuFEx) ~ 10^−2^ s^−1^) were differentially affected by the presence of a common affinity ligand (*k*_*inact*_ (acylimidazole) ~ 10^−5^ s^−1^, *k*_*inact*_ (NASA) ~ 10^−5^ s^−1^ and *k*_*inact*_ (SuFEx) ~ 10^−4^ s^−1^)^111^. Some of the aforementioned challenges for transfer t-LDL could be mitigated by a wider understanding and systematic comparison of warhead selectivity and kinetics across multiple model proteins, but such comparisons are currently limited. Whilst labelling yield is frequently reported and some studies on the intrinsic reactivity of warheads are available, translating these to different targets and biological contexts remains underexplored. Nonetheless, the rapid labelling that can be achieved with certain warheads e.g. NASA series (*k*_*L*_ up to ~ 10^−3^ s^-1^ and *k*_*L*_/*K*_*d*_ up to ~ 3 ×10^4^ M^−1^s^−1^) is on a timescale that would permit mechanistic analyses of temporal biological processes (e.g. mitosis ~ 30-60 mins).

### Catalyst-tethered t-LDL

**Fig. 9:**
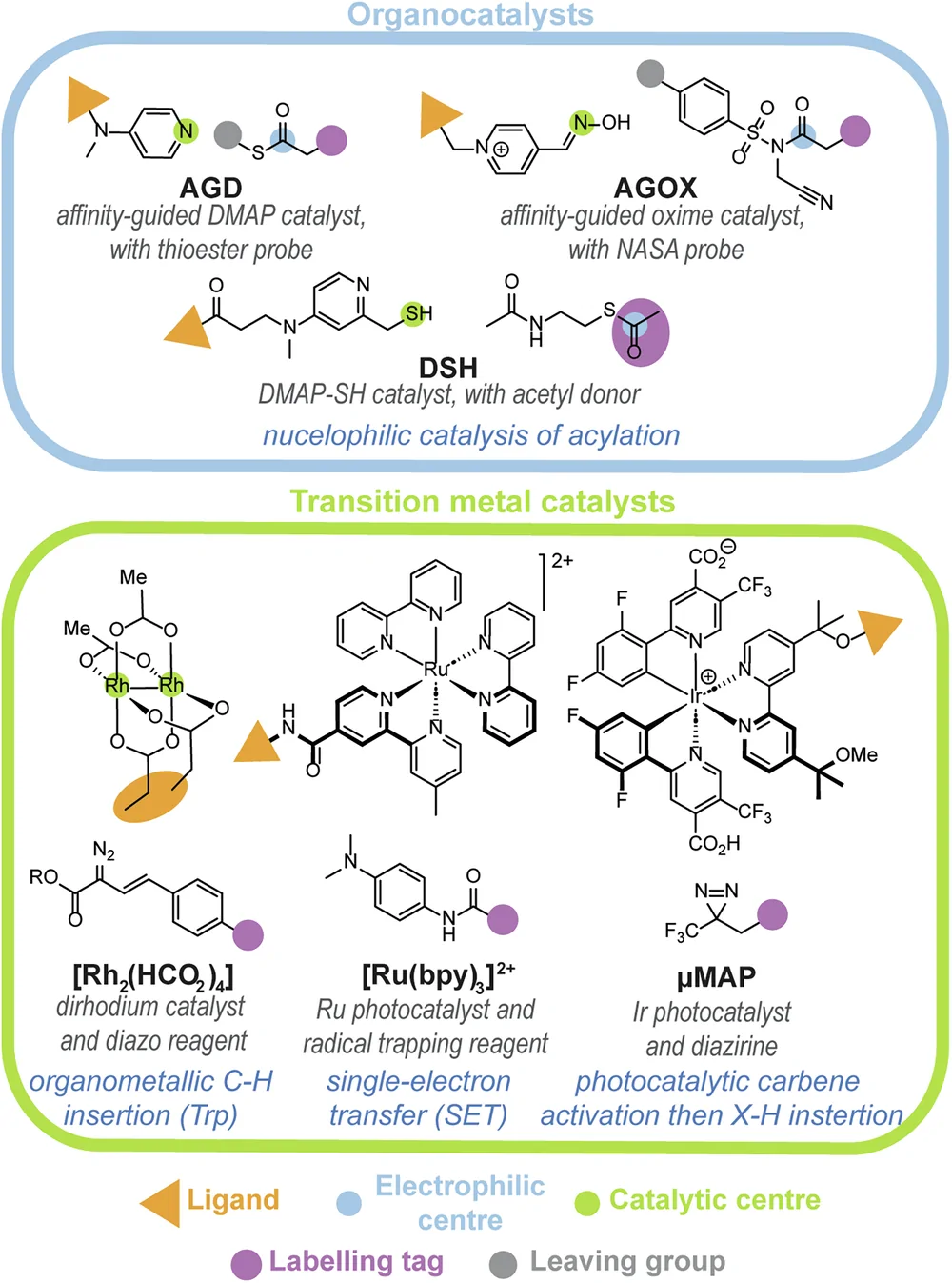
Examples of catalyst-tethered t-LDL systems. Generalised structures of catalyst t-LDL reagents. For organocatalysts, these comprise a nucleophile connected to the POI ligand, and a separate electrophilic tag or labelling group bearing the reactive centre; for transition metal (TM) catalysts, these comprise a TM catalyst connected to the POI ligand, and a separate reactive tag or labelling group.

Catalyst-tethered t-LDL, while mechanistically distinct, has developed in parallel with transfer t-LDL. Some catalyst-tethered systems closely mirror transfer t-LDL warheads, such as the organocatalysts pioneered by the Hamachi group; others exploit different reagents and mechanisms of action, such as the photocatalytic approaches developed by the Nakamura and MacMillan groups (Fig. 9).

### Organocatalytic t-LDL

The Hamachi group introduced early examples of catalyst-tethered LD chemistry with their work on affinity-guided DMAP (AGD) probes. These probes were first reported in 2007 and designed to label sugar-binding lectins by tethering a catalytic DMAP group to a sugar recognition motif. Subsequently, feeding in an acyl donor allowed the DMAP to catalyse an acyl transfer reaction to label a nucleophilic residue on the target lectin^112^. AGD chemistry initially experienced several limitations: the chemistry was sensitive to modification of the acyl donor structure, the acyl donor probes were susceptible to hydrolysis, which made the chemistry unsuitable for intracellular work, and the chemistry was slow, often requiring long incubation periods under basic conditions (pH 8) to achieve labelling^112,113^. Despite these challenges, AGD chemistry has been successfully applied to labelling membrane proteins and cell-surface glycoproteins in live cells^114,115^, demonstrating its potential. In 2014, improved understanding of the reactivity of the acyl donor enabled the first example of intracellular AGD labelling. In this example from Song et al., reducing the innate reactivity of the acyl donor and accommodating this by moving to a multi-catalyst motif yielded a system capable of labelling FKBP12 *in cellulo*^113^.

**Fig. 10:**
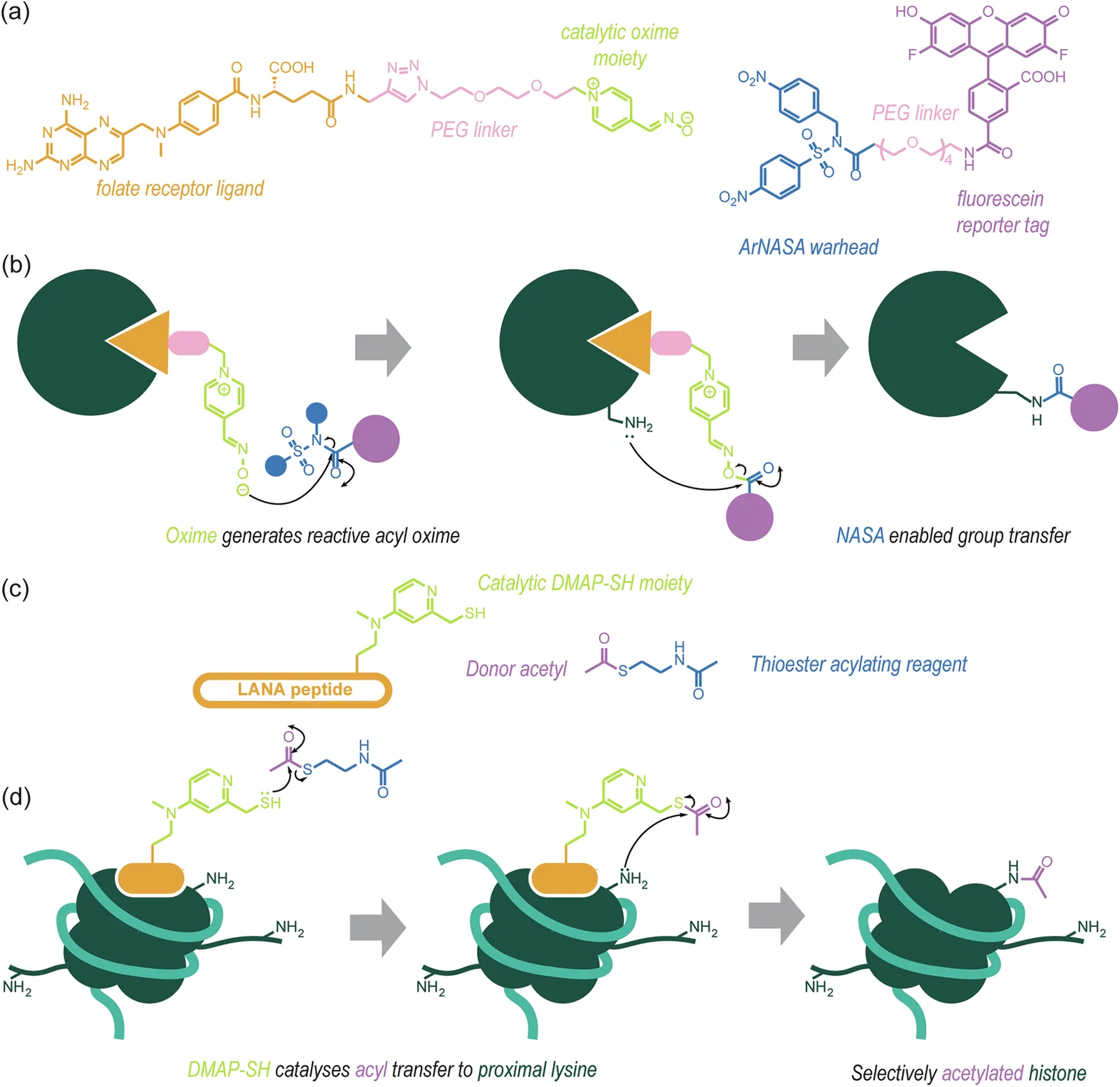
Organocatalytic t-LDL. **a** Structure of AGOX/ArNASA probe utilised by Tamura et al.; **b** Schematic illustration of AGOX t-LDL; **c** Structure of DMAP-SH probe and complementary thioester reagent utilised by Fujiwara et al.; **d** Schematic illustration of DMAP-SH enabled histone acetylation.

A further innovation has been the development of a catalyst-tethered approach utilising the NASA warhead. Here, affinity-guided oxime (AGOX) chemistry is a mechanistically similar successor to the earlier AGD chemistry and improves upon several of the limitations displayed by the earlier AGD chemistry. Replacing the DMAP catalytic motif with the more nucleophilic pyridinium oxime motif shifts some of the burden of reactivity from the acyl donor probe to the catalytic motif (second order rate constants (*k*_2_) of 2.14 ± 0.08 M^–1^ s^–1^ for hydrolysis of *p*-nitrophenolacetate by an oxime and 0.237 ± 0.001 M^–1^ s^–1^ for DMAP emphasise the superior intrinsic reactivity); this allows thioester-based acyl donors, with their high intrinsic electrophilicity and susceptibility to hydrolysis and esterases, to be swapped for NASA-based acyl donors. The resultant AGOX system (Fig. 10a, b) has proven more capable of labelling challenging targets; for example, AGOX chemistry was successfully applied to label AMPAR in brain tissue, demonstrating its ability to function in complex tissue environments^116^. Raynal et al. recently demonstrated that this approach could be used in the context of a peptide-based ligand to effect protein labelling with a broad range of NASA-based acyl donors, broadening its scope to peptide-based libraries^117^.

The Kanai group developed a DMAP-SH (DSH) based catalyst which, when tethered to a suitable ligand, and together with a suitable thioester acylating reagent, could be used to acylate histones in live cells (Fig. 10c, d)^118,119^. A hydroxamic acid–piperidine conjugate could also be used^120^, and subsequently, the thioester acylating reagent could be substituted with native acetyl-coenzyme A^118,119^.

Organometallic catalysis has also been used for catalyst-tethered t-LDL. Ball and coworkers used dirhodium tetracarboxylate-based catalysts to effect C–H insertion reactions of tryptophan residues using diazo-based alkylating agents^121,122^. These studies were carried out initially using coiled-coils, and later applied to antibody functionalisation^123^. In addition, a series of small molecules, which act on STAT3 function, were functionalised with a dirhodium tetracarboxylate catalyst and used to identify the ligand binding site in the coiled-coiled domain of STAT3^124^.

### Photocatalytic t-LDL

**Fig. 11:**
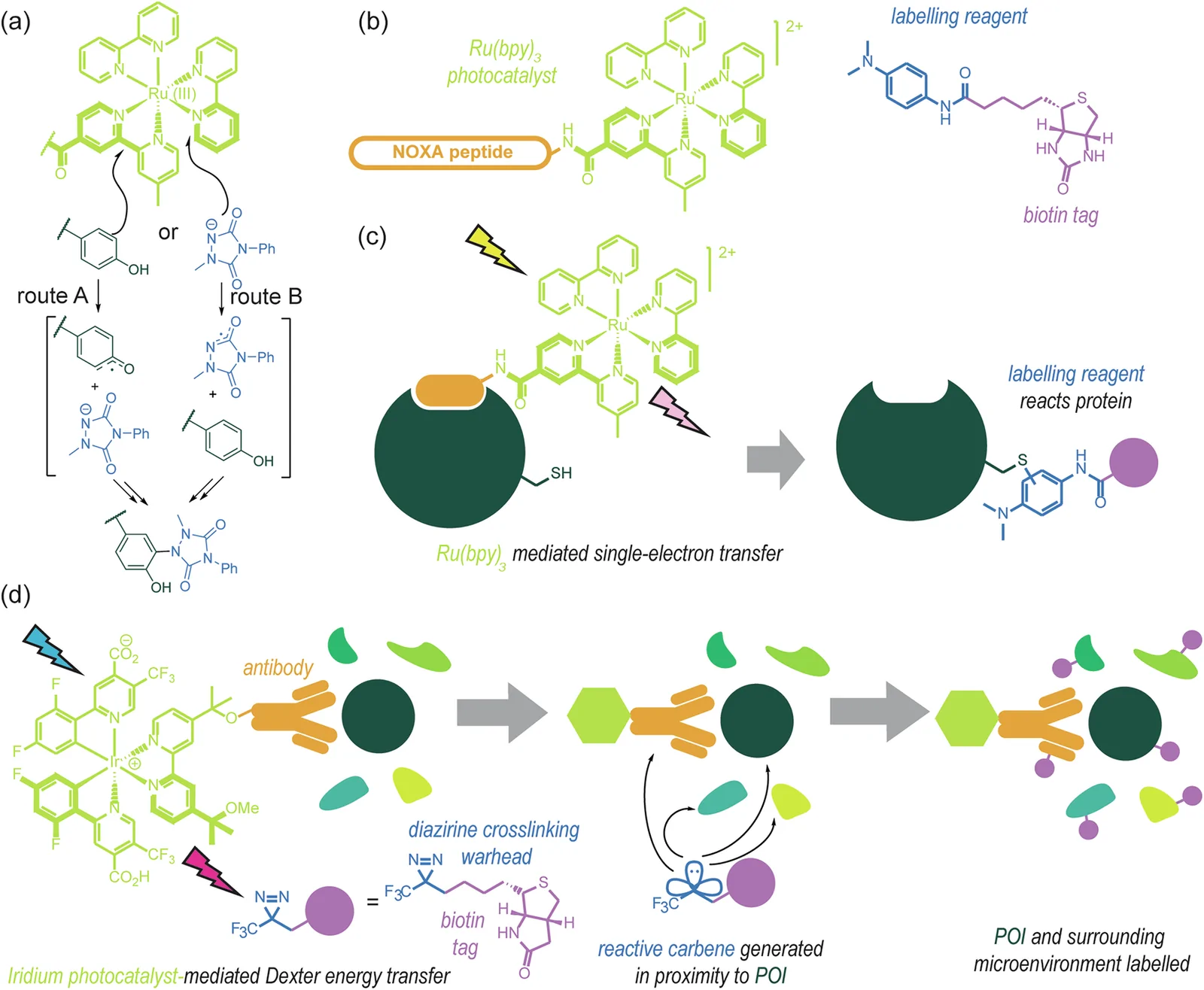
Photocatalytic t-LDL. **a** illustrative mechanistic pathways for [Ru(bpy)_3_]^2+^ mediated protein labelling; following photoexcitation to a Ru(III) species, radical formation can occur on protein residues (e.g., Tyrosine) or the labelling reagent prior to trapping and covalent bond formation; **b** Structure of [Ru(bpy)_3_]^2+^ probe utilised by Beard et al.; **c** Schematic illustration of [Ru(bpy)_3_]^2+^ t-LDL; **d** Structure of µMap probe developed by Geri et al.; alongside schematic illustration of µMap t-LDL.

Photocatalytic labelling represents an alternative approach to t-LDL and can take two forms: in the first approach, a ligand-tethered photocatalyst is used to activate an amino acid residue on the POI, which then traps a suitably functionalised tag; in the second, the photocatalyst is used to activate the tag, which then traps a suitably reactive residue on the target protein. Ru(bpy)_3_-mediated single-electron transfer (SET) has emerged as a promising approach for catalyst-tethered t-LDL. This approach emerged from work by the Kodadek group, who first reported the crosslinking of proteins using the photocatalytic properties of [Ru(bpy)_3_]^2+^ under oxidative conditions over twenty years ago. This approach was applied to the crosslinking of UvsY, a hexamer involved in phage T4 recombination, alongside homodimeric and tetrameric transcription factors. The proposed mechanism involves persulfate-mediated oxidation of the photo-excited state, [Ru(bpy)_3_]^2+^*, to generate [Ru(bpy)_3_]^3+^ and a sulphate radical. SET from a tyrosine residue to the [Ru(bpy)_3_]^3+^ results in a tyrosyl radical which reacts with a proximal nucleophile (tyrosine, tryptophan, methionine or cysteine), followed by hydrogen abstraction by the sulphate radical^125^. In 2013, Sato and Nakamura reported that Ru(bpy)_3_ appended to a suitable ligand could be used to effect labelling of carbonic anhydrase with *N*′-acetyl-*N*,*N*-dimethyl-1,4-phenylenediamine tagging reagents^126^. The method was subsequently applied to labelling EGFR in cells, albeit with off-target reactivity and oxidative degradation of the protein^127^. In both cases, it may have been that some labelling arose from generation of the radical on the trapping reagent and subsequent reaction with tyrosine, as opposed to generation of a tyrosyl radical. This appeared to be confirmed by switching the *N*′-acetyl-*N*,*N*-dimethyl-1,4-phenylenediamine tagging reagent for 1-methyl-4-aryl-urazole^128^, which forms a shorter-lived radical and in effect leads to a shorter labelling radius ( ~ 1.4 nm dictated by the proximity between the Ru(bpy)_3_ and tyrosine residue). Finally, in 2019 Beard et al. appended a BH3 peptide mimic with a Ru(bpy)_3_ warhead, producing LDL probes capable of selectively labelling MCL-1 (Fig. 11a, b)^129^. In this instance, a cysteine was preferentially labelled. The temporal resolution and specificity offered by these probes demonstrated their potential for studying PPIs. The approach has also been used to induce protein inactivation by generation of reactive oxygen species (ROS)^130^.

In 2020, the groups of Macmillan and Muir introduced methodology for photocatalytic proximity labelling that excites the tagging reagent (Fig. 11c, d). Using an iridium-based photocatalyst, it was shown that excitation in the presence of diazirines led to carbene formation and subsequent labelling of BSA. Subsequently, using iridium-tethered antibodies for bead-adsorbed receptors, e.g., VEGFR2, and cell-surface receptors, e.g., CD45, CD47, CD29, and PD-L1, it was shown that proximal proteins could be tagged with the carbene resulting from diazirine excitation^131^. It was proposed that this method – µMap, operates via Dexter energy transfer from the photocatalyst to the diazirine, resulting in a shorter labelling radius (diazirine half-life (*t*_1/2_) ~ 2 ns < 4 nm labelling radius) and superior spatiotemporal resolution to other techniques such as BioID and APEX (*t*_1/2_ 0.1 ms to 60 s ~ 10-20 nm labelling radius)^11,131^. This approach has been rapidly broadened to small molecule-tethered photocatalysts to establish drug and binding site identification^132,133^, catalysts that permit labelling using nitrenes (*t*_1/2_ = ~ 10 ns)^134,135^ or sulfonium diazo labels^136^ and HaloTag/chloroalkane-appended iridium photocatalysts^137^, analyses of the interactome of aggregation-prone proteins^138^, alongside a range of mechanistic and discovery analyses of biological processes^139,140^. Further recent development of the approach has been reported by Lin et al. Their MultiMap approach uses a single photocatalyst alongside a range of photo-probes with varying radii to capture both short- and long-range proximity labelling to yield multiscale information on interactomes^141^.

It is also possible to use an organic cofactor *in lieu* of the co-ordination complex to effect photoproximity labelling^142^. Referred to as deazaflavin–diazirine energy-transfer labelling (DarT labelling), this approach has been used in conjunction with the HaloTag/chloroalkane fusion system and through direct conjugation to cyclic cell-penetrating peptides to map interactomes. Photocatalysts that localise to specific organelles e.g., the mitochondria, can also label proteins localised to that organelle using nitrenes^143^. Finally, Scinto et al. recently reported a system that leverages the biorthogonal tetrazine ligation to effect photocatalytic t-LDL rather than highly reactive intermediates such as carbenes or nitrenes, allowing for greater fine-tuning of labelling radius^144^.

### Summary of catalyst-tethered t-LDL chemistries

Catalyst-tethered t-LDL has developed parallel to transfer t-LDL but has evolved a different set of strengths and applications. Catalyst-tethered t-LDL has emerged as a powerful supplement to the organic warheads of transfer t-LDL. The advantage offered by separating the ligand from the reporter tag has been demonstrated by Raynal et al.’s work on developing a highly modular approach for modification of target proteins with a variety of functionalities and Fujiwara et al.’*s* impressive application of organocatalyst-tethered t-LDL by exploiting the flexibility of the chemistry to synthetically introduce PTMs to native histones in live cells^117,118,120^.

The most notable difference between transfer t-LDL and catalyst-tethered t-LDL is in the use of photocatalysts. These systems have evolved to rely less upon chemoselectivity and instead leverage general proximity to enable new applications. This is exemplified by the photocatalytic t-LDL reagents developed by the Nakamura and MacMillan groups^126,127,131^, the µMap approach in particular relies almost exclusively on proximity due to the highly reactive nature of the carbenes generated. This shift has opened the door to new applications of t-LDL such as exploiting the general proximity labelling for highly spatiotemporally controlled interactome mapping^133,137,139,140^. However, catalyst-tethered t-LDL is not without its challenges; organocatalyst-tethered t-LDL chemistries remain difficult to optimise due to high off-target reactivity. Photocatalyst-tethered t-LDL offers unique reactivity that can be exploited for new and distinct applications, but relies on and reports on proximity rather than direct physical interaction. As a result, transfer t-LDL and catalyst-tethered t-LDL represent complementary approaches.

### Outlook

In the last decade, t-LDL chemistry has emerged as a promising tool for chemical biology. It is an exciting technology for two primary reasons: it offers the potential to study native proteins in their endogenous environment, and probes can be designed from previously identified ligands and inhibitors^19^. The ability to utilise previously identified ligands as scaffolds allows researchers to draw on the results of extensive chemical probe and drug discovery programmes. Several groups have utilised the products of such research as probes for further investigation of complex biological systems or as tools for developing new platforms for ligand discovery^20,47,52^.

The scope of t-LDL chemistries has expanded significantly in recent years. The number of t-LDL-suitable warheads has increased, which has resulted in more specific and tuneable warheads, the ability to target a greater range of residues, and more diverse mechanisms of action. In particular, the NASA/ArNASA warhead is an example of a well-functioning t-LDL warhead, which has been adopted by a range of groups to study challenging targets. Also notable are the methacrylamide and SuTEx warheads, which allow t-LDL chemistry to be performed at cysteine and tyrosine, respectively, which significantly expand the scope of LDL. Complementary, catalyst-tethered LDL chemistries such as µMap harness the unique capabilities of synthetic organic chemistry to allow rapid labelling across a narrow radius for interactome analyses. These advances, in combination with the depth of literature regarding small-molecule protein ligands, pave the way for t-LDL to become a powerful technology for research; however, t-LDL chemistry still requires further innovation. There are a number of outstanding questions and ongoing technical challenges, as demonstrated by the continued iteration and improvement of t-LDL chemistries. While selectivity is driven by both ligand specificity and warhead chemoselectivity, the relationship between these has not been comprehensively studied; the timescale of labelling and the longevity of tags within cells remain underexplored, and the downstream effects of labelling, particularly with complex/functional tags, warrant further characterisation. Further thorough investigation as to how post-labelling ligand rebinding may affect the target protein is also needed; this remains a crucial question where t-LDL probes are based on known inhibitors that have pronounced effects on complex biological systems.

The power of t-LDL is starting to be exemplified. A number of groups have utilised t-LDL probes to covalently attach a bioorthogonal reactive handle at a target site, which is then used in conjunction with fluorescent tags for imaging or pull-down assays; others have exploited the covalent transfer of the adduct group for proteomics studies. Another common, but mechanistically different, application of t-LDL chemistry is to use the leaving group as a ‘turn-on fluorescence’ probe for a quantitative readout for inhibition of the target POI. Finally, a notable trend in recent years has been the emergence of LDL probes that transfer secondary functional groups to the target protein (e.g., PHICS discussed above) or synthetically mimic post-translational modification of a target. This ‘rewiring’ modality offers powerful tools for exploring PPIs and may have value in therapeutic applications. Overall, these developments promise a bright future for t-LDL.

## Supplementary information

Transparent Peer Review file

## Data Availability

There is no data associated with this review.
