# Supplementary material for: Progress and challenges in traceless ligand-directed labelling chemistry
Source: Commun Chem. 2026 Sep 29;9:310. doi: 10.1038/s42004-026-02222-0 (PMC13623825; doi:10.1038/s42004-026-02222-0)
Supplement: Supplementary file 1 [file 42004_2026_2222_MOESM1_ESM.pdf]

nature portfolio

Peer Review File

Progress and Challenges in Traceless Ligand-Directed Labelling Chemistry

Corresponding Author: Professor Andrew Wilson

This file contains all reviewer reports in order by version, followed by all author rebuttals in order by version.

Version 0:

Reviewer comments:

Reviewer #1

(Remarks to the Author)
The authors argue that “traceless ligand-directed labeling (t-LDL) chemistry” is a powerful approach for understanding the molecular mechanisms of protein–protein interactions that regulate diverse cellular functions, because it enables endogenous proteins to be labeled without impairing their function. Specifically, t-LDL chemistry allows labeling at a site distant from the active center of the protein, and the protein ligand used for labeling dissociates after the labeling reaction, thereby minimizing perturbation of protein function.
The importance of traceless chemical labeling is evident from the fact that, as also described in this manuscript, many researchers have recently been developing a variety of approaches in this area. In recent years, applications have expanded beyond intracellular use to in vivo studies in mice and other biological systems. Therefore, a review on traceless chemical labeling would likely be of interest to a broad range of researchers.
However, several reviews on related topics, including more comprehensive ones, have been published within the past one to two years. Examples include the following. To clarify the positioning of the present manuscript and to distinguish it from previously published reviews, these articles should at least be appropriately cited.
”Covalent lysine targeting is coming of age” by London, N. et al., https://doi.org/10.1016/j.cbpa.2026.102671; “Advancing Covalent Ligand and Drug Discovery beyond Cysteine” by Hsu, K.-L. et al., https://doi.org/10.1021/acs.chemrev.5c00001; “Targeted Covalent Modification Strategies for Drugging the Undruggable Targets” by Tamura, T. and Hamachi, I. et al., https://doi.org/10.1021/acs.chemrev.4c00745.
Furthermore, although the manuscript classifies various labeling methods according to the type of labeling reaction and the target amino acid residues, the information provided is somewhat qualitative. As stated in the Outlook section, the selectivity for labeled amino acid residues and the reaction kinetics vary considerably among different methods, and these aspects remain important areas for future improvement. Therefore, to help readers gain a deeper understanding of these points, it would be useful to summarize, for each method, the selectivity for labeled amino acid residues, the labeling ratio for each amino acid if available, and kinetic parameters in a table.
It may also be helpful to include a short summary paragraph at the end of each major section, such as Section 4. Transferase t-LDL and Section 5. Catalyst-tethered t-LDL, to help readers better organize the content.
Another point of concern is that the authors classify t-LDL into two categories, one of which is termed “transferase LDL.” I have some concerns about the appropriateness of this terminology. It is understandable in the sense that the labeling tag is transferred from the protein ligand to an amino acid residue on the target protein. However, the term “transferase” may give the impression that the reaction proceeds enzymatically or catalytically. In most cases, however, this is presumably not the case; rather, one molecule of the labeling substrate labels one amino acid residue, and further labeling would require an additional molecule of the labeling substrate. If this understanding is correct, the use of the term “transferase LDL” should be reconsidered.

Minor points are as follows:
1. p. 2, l. 43: “SNAP” should be written as “SNAP-tag.”
2. p. 3, l. 71: “HALO tag” should be written as “HaloTag.”
3. p. 33, l. 972: The title of Ref. 115 appears to be garbled.
4. p. 34, l. 1016: Ref. 130 and Ref. 131 appear to be duplicates of Ref. 38 and Ref. 43, respectively.

Reviewer #2

(Remarks to the Author)
The review article titled “Progress and Challenges in Traceless Ligand-Directed Labelling Chemistry” authored by Toby Hopkinson et al. presents recent chemical techniques used in ligand-directed traceless labelling methods. Although many reviews have previously been published on similar topics, the authors provide a concise report of the most recent advances in this field with notable applications. In view of this, I recommend the manuscript for publication after minor revisions.
1. In the Introduction section, the authors clearly describe the genetic approaches for protein labelling. Figure 1 also illustrates these approaches; however, the figure appears somewhat confusing as the explanation relies mainly on colour coding. I recommend a clearer and more informative representation of the figure.
2. There appears to be a typo/error in the structure shown in Figure 8B at the POI-labelled histidine residue, where one carbon atom seems to be missing.

Reviewer #3

(Remarks to the Author)
The authors have selected an interesting topic for this review. Achieving selective chemical modification within complex biological systems while minimizing permanent perturbation of protein structure and function remains a significant challenge. Hence, an overview of current methods, highlighting their strengths and limitations, could assist in the development of new platforms for traceless labeling of proteins and the cellular system.
Unfortunately, it presents an account rather than reviewing the topic in most cases. The figures have considerable scope for improvement and can benefit the quality of the manuscript. While the write-up provides a good collection of relevant examples, the figures don't do justice to the sections affecting the readability. Besides, the mechanistic discussion has been skipped for many cases. A few examples (covalent inhibitors) included in the manuscript do not fully align with the main theme of the review highlighted in the introduction. Importantly, the gaps or unsolved technical challenges, while recognized by the community, have not been discussed in this manuscript.

A few representative points are highlighted below to assist in the process.

1. The abstract and introduction highlight protein-protein interaction. The discussion in the follow-up text completely ignores it. The focus shifts predominantly to the warheads and chemistry available for traceless labeling. There is a disconnect between the anticipation set by the introduction and the main content.

2. The introduction primarily focuses on genetic approaches for protein labeling and their associated limitations. Ligand-free chemical methods for protein modification and their associated challenges are essential for setting the context for selectivity attributes enabled by ligands. Including a brief discussion of these approaches would better clarify the limitations of nonspecific chemical labeling strategies and more effectively highlight the need for ligand-directed labeling chemistry.

3. Page 4, line 112: The described strategy for designing probes targeting residues away from the protein active site appears conceptually similar to the approaches commonly used in covalent inhibitor design that target active-site residues. This part needs more discussion for clarity. Additionally, the role of the linker is particularly important in enabling access to residues located away from the active site, and could be discussed.

4. Figure 2b: A reducing agent is used to cleave the disulfide bond between the ligand and the probe. The authors emphasize chemical methods for protein labeling without disturbing protein structure; however, the reducing conditions employed may also reduce native disulfide bonds present within proteins, potentially affecting their structural integrity and biological function. A disclaimer for such cases, highlighting the limitations, is needed.

5. Figure 3b: JQ1 is represented as a labeling tag; however, JQ1 is a well-known ligand for BET family proteins. The authors may consider clarifying their role.

6. Figure 3c: C–S–C linkage - bond angle needs correction.

7. Figures 3 and 4: While the authors compile various warheads, including the operational mechanism or mode of action, would add clarity.

8. Figure 5: The overall illustration needs more clarity. The arrow pushing should initiate from the lone pair of the amine. The lemon-coloured component should be properly labeled to indicate its role in the system. In addition, the inclusion of UV irradiation for diazirine activation would provide mechanistic clarity.

9. Page 10, line 248: The authors discuss KHAc, a derivative of a PGAM1 inhibitor, which may potentially alter the native activity of the target protein. Is there a pressing reason to keep this example, as it does not align with the arguments in the introduction?

10. Page 11, line 260: The authors state that “the SEAlide is unreactive in the absence of the protein target.” What is the extent of selectivity? Does it imply exclusive selectivity towards the POI?

11. Page 11: The discussion related to reference 66 appears to involve a covalent inhibitor-based strategy. The authors should reconsider including this example or explain its relevance in the context of tracelessness.

12. The authors discuss the first- and second-generation NASA-based LDL strategies; however, inclusion of a comparative figure illustrating the chemical modifications between these could help understand the structural changes that influence selectivity and reactivity.

13. Figure 7c: The bonds are not drawn properly for iminoboronate.

14. Figure 9: The authors primarily present the catalysts and reactive warheads; however, inclusion of the corresponding reaction mechanism would add clarity.

15. Authors discuss the chemistry presented in references 102 and 116 in considerable detail; the inclusion of a representative figure/mechanism for these examples would add value.

Other minor comments:
1. There are a few editing errors in the draft that require proofreading.
2. The authors may consider numbering the subtitles for Lysine-targeting warheads as
4.2.1 Sulfonylation
4.2.2 Acylation
3. Reference 90 doi: not available

Version 1:

Reviewer comments:

Reviewer #3

(Remarks to the Author)
I believe the authors need to further refine the manuscript to be considered favorably.
Here are a few suggestions:
[1] The authors state that a discussion of ligand-free protein labeling has been added to Section 1.1; however, I am unable to trace it in the revised manuscript.
[2] The C–S–C bond angle in Figure 3c appears to remain unchanged and still seems incorrect.
[3] Figure 10b: Final product C–N–Carbonyl linkage - bond angle needs correction.
[4] The authors' response states that pertinent mechanistic details have been included in Figures 9 and 10; however, I do not see them in Figure 9 of the revised manuscript. The figure continues to present the catalysts and reactive warheads primarily, without indicating their corresponding modes of action.

Open Access This Peer Review File is licensed under a Creative Commons Attribution 4.0 International License, which

permits use, sharing, adaptation, distribution and reproduction in any medium or format, as long as you give appropriate credit to the original author(s) and the source, provide a link to the Creative Commons license, and indicate if changes were made.
In cases where reviewers are anonymous, credit should be given to 'Anonymous Referee' and the source.
The images or other third party material in this Peer Review File are included in the article's Creative Commons license, unless indicated otherwise in a credit line to the material. If material is not included in the article's Creative Commons license and your intended use is not permitted by statutory regulation or exceeds the permitted use, you will need to obtain permission directly from the copyright holder.
To view a copy of this license, visit https://creativecommons.org/licenses/by/4.0/

School of Chemistry
University of Birmingham
Edgbaston, Birmingham, B15 2TT, United Kingdom
a.j.wilson.1@bham.ac.uk

Date: 5th August 2026
Subject: COMMSCHEM-26-0366.R1

Dear Editor,

Please find attached a revised review article, *"Progress and Challenges in Traceless Ligand-Directed Labelling Chemistry"* by Hopkinson, *et al.*, which we hope is now considered acceptable for publication as a review in the Nature Portfolio Collection on "*Chemical modification of proteins*", specifically *Communications Chemistry*. Apologies for the delay in returning this revision to you. We submitted the original version of the review following discussion with Dr Guo who agreed to consider the paper and to apply a full fee waiver should it be accepted. We are grateful to the reviewers for their comments and have addressed them with changes to the manuscript highlighted yellow (Figures 1-8 have also been revised). We have also added discussion of and cited additional papers published during reviewing and identified as relevant during the revision alongside minor typographical issues also highlighted yellow. Our specific responses are as follows:

Reviewer #1

'Several reviews on related topics, including more comprehensive ones, have been published within the past one to two years. Examples include the following. To clarify the positioning of the present manuscript and to distinguish it from previously published reviews, these articles should at least be appropriately cited. "Covalent lysine targeting is coming of age" by London, N. et al., https://doi.org/10.1016/j.cbpa.2026.102671; "Advancing Covalent Ligand and Drug Discovery beyond Cysteine" by Hsu, K.-L. et al., https://doi.org/10.1021/acs.chemrev.5c00001; "Targeted Covalent Modification, Strategies for Drugging the Undruggable Targets" by Tamura, T. and Hamachi, I. et al., https://doi.org/10.1021/acs.chemrev.4c00745.'

Indeed there are a significant number of reviews on protein covalent labelling chemistry, including those suggested by the reviewer, however, the scope of our review is different from these recent reviews as it focuses specifically on t-LDL chemistry (including the most recent developments) rather than covalent ligand discovery or recent developments focusing on the labelling of a specific residue. In our original submission, we included those reviews which we considered were sufficiently broad and comprehensive to direct the reader to the key relevant concepts of covalent protein chemistry but have added those suggested by the reviewer, alongside 10.1038/s41573-022-00542-z

'.....although the manuscript classifies various labeling methods according to the type of labeling reaction and the target amino acid residues, the information provided is somewhat qualitative. As stated in the Outlook section, the selectivity for labeled amino acid residues and the reaction kinetics vary considerably among different methods, and these aspects remain important areas for future improvement. Therefore, to help readers gain a deeper understanding of these points, it would be useful to summarize, for each method, the selectivity for labelled amino acid residues, the labeling ratio for each amino acid if available, and kinetic parameters in a table.'

and

'....helpful to include a short summary paragraph at the end of each major section, such as Section 4. Transferase t-LDL and Section 5. Catalyst-tethered t-LDL, to help readers better organize the content.'

UNIVERSITY OF BIRMINGHAM

The information provided is necessarily qualitative and we note that the lack of systematic comparison between warheads presents an outstanding question for t-LDL. While some characterisation of labelling is available in the literature – labelling yield is consistently reported, for example, and some authors report studies on the intrinsic reactivity of the warheads, other data such as ratio of different amino acids labelled, quantitative on/off target reactivity and rate of labelling in biological contexts is much more inconsistently reported. Furthermore, the reliance of t-LDL on proximity-assisted rate enhancement makes the comparison between model proteins difficult and complicates the use of intrinsic reactivity studies as a metric for warhead performance. We have added commentary to this effect, details on selected warheads/chemistries (such as kinetics of target labelling and labelling radius) and a reference describing a recent study on covalent inhibitors highlighting the environment dependence of labelling chemistry (10.1021/acscentsci.5c00699). *In lieu* of a tabulated comparison we have added summaries at the end of Section 4, Transfer t-LDL, Section 5, and Catalyst-tethered t-LDL, which now include a discussion of these factors as well as highlighting the limitation of making such comparisons and opportunities for future research.

'.....the authors classify t-LDL into two categories, one of which is termed "transferase LDL." I have some concerns about the appropriateness of this terminology. It is understandable in the sense that the labeling tag is transferred from the protein ligand to an amino acid residue on the target protein. However, the term "transferase" may give the impression that the reaction proceeds enzymatically or catalytically. In most cases, however, this is presumably not the case; rather, one molecule of the labeling substrate labels one amino acid residue, and further labeling would require an additional molecule of the labeling substrate. If this understanding is correct, the use of the term "transferase LDL" should be reconsidered.

We thank the reviewer for this valid point. We have amended the manuscript to replace the term, 'transferase,' with 'transfer LDL' or 'transfer chemistry' where appropriate to avoid any suggestion that the reaction is enzyme-catalysed.

Minor points are as follows:

1. p. 2, l. 43: "SNAP" should be written as "SNAP-tag."
2. p. 3, l. 71: "HALO tag" should be written as "HaloTag."
3. p. 33, l. 972: The title of Ref. 115 appears to be garbled.
4. p. 34, l. 1016: Ref. 130 and Ref. 131 appear to be duplicates of Ref. 38 and Ref. 43, respectively.

These typographical and formatting errors have been corrected in our revised manuscript.

Reviewer #2

'...the authors clearly describe the genetic approaches for protein labelling. Figure 1 also illustrates these approaches; however, the figure appears somewhat confusing as the explanation relies mainly on colour coding. I recommend a clearer and more informative representation of the figure.'

We have reworked Fig. 1 to provide more clarity to the discussion of genetic approaches to protein labelling: the labelling within the figure has been expanded, figure subtitles made clearer and a legend has been added to clarify the colour coding.

'......typo/error in the structure shown in Figure 8B at the POI-labelled histidine residue, ...one carbon atom seems

to be missing.'

Figure 8 has been amended to include the additional carbon atom of the histidine residue.

Reviewer #3

'Unfortunately, it [the review] presents an account rather than reviewing the topic in most cases. The figures have considerable scope for improvement and can benefit the quality of the manuscript. While the write-up provides a good collection of relevant examples, the figures don't do justice to the sections affecting the readability. Besides, the mechanistic discussion has been skipped for many cases. A few examples (covalent inhibitors) included in the manuscript do not fully align with the main theme of the review highlighted in the introduction. Importantly, the gaps or unsolved technical challenges, while recognized by the community, have not been discussed in this manuscript.'

We do not consider that it is necessary to include a detailed mechanistic discussion of the labelling chemistries as these are generally established (see later specific responses), rather the challenges in ensuring fast, selective and (depending on the purpose) side chain specific labelling of target proteins. This relates to the reactivity of the warheads (e.g. electrophilic warheads, will have some reactivity towards all of the nucleophilic side chains), affinity of the ligand for the target protein, quantitative on/off target reactivity, local environment (incl. pH) and rate of labelling in biological contexts, nature/effect of any linker between protein-ligand and reactive group/catalyst, effect of labelling on protein structure and function (see also response to reviewer 1). This relates to the second key point i.e. absence of discussion of challenges recognized by the community. We also perceive that for t-LDL these challenges exist, but the current status of the field is the data highlighting these issues is not well-documented in the published literature (or conference proceedings); one rare example is: https://doi.org/10.1002/anie.202512131 where the NASA warhead was explicitly shown to exhibit promiscuous reactivity towards several side chains on the target protein (which we highlighted). In our revised manuscript we have endeavoured to better draw out what we consider these challenges to be whilst ensuring any critical analyses are evidence based.

' The abstract and introduction highlight protein-protein interaction. The discussion in the follow-up text completely ignores it. The focus shifts predominantly to the warheads and chemistry available for traceless labeling. There is a disconnect between the anticipation set by the introduction and the main content.'

We have amended the abstract to more clearly reflect the reviews' discussion and showcasing of t-LDL chemistry. The introduction has also been reworked to have less focus on protein-protein interactions and instead discuss genetic and chemical approaches to labelling proteins to better set up the discussion of ligand-directed chemistry and specifically t-LDL in the review's main content.

'The introduction primarily focuses on genetic approaches for protein labeling and their associated limitations. Ligand-free chemical methods for protein modification and their associated challenges are essential for setting the context for selectivity attributes enabled by ligands. Including a brief discussion of these approaches would better clarify the limitations of nonspecific chemical labeling strategies and more effectively highlight the need for ligand-directed labeling chemistry.'

We have reworked the introduction (see above) and also integrated a discussion of ligand-free protein labelling in the introduction to better highlight the need for ligand directed chemistry renumbered section 1.1.

UNIVERSITY OF BIRMINGHAM

'Page 4, line 112: The described strategy for designing probes targeting residues away from the protein active site appears conceptually similar to the approaches commonly used in covalent inhibitor design that target active-site residues. This part needs more discussion for clarity. Additionally, the role of the linker is particularly important in enabling access to residues located away from the active site, and could be discussed.

We have added explicit reference to the linker as a fundamental aspect of t-LDL probe design and highlighted the parallels to covalent inhibitor development.

'Figure 2b: A reducing agent is used to cleave the disulfide bond between the ligand and the probe. The authors emphasize chemical methods for protein labeling without disturbing protein structure; however, the reducing conditions employed may also reduce native disulfide bonds present within proteins, potentially affecting their structural integrity and biological function. A disclaimer for such cases, highlighting the limitations, is needed.'

We have included this additional detail and highlighted the limitations of the approach. We also included additional context that the strategy depicted in Fig 2b was an early example which provided motivation for further development of t-LDL.

' Figure 3b: JQ1 is represented as a labeling tag; however, JQ1 is a well-known ligand for BET family proteins. The authors may consider clarifying their role.'

Figure 3b and the associated discussion on page 7 have been amended to clarify that while the target protein is being 'tagged' with JQ1, JQ1 is independently a ligand for a second family of proteins, namely BET proteins.

' Figure 3c: C–S–C linkage - bond angle needs correction.'

The C-S-C bond angle in Figure 3c has been corrected.

'Figures 3 and 4: While the authors compile various warheads, including the operational mechanism or mode of action, would add clarity.'

The mechanisms of the reactions used for labelling in these figures are conventional, well established, and we consider, will be understood by the readers. We have included mechanistic details in figures of those reactions that are more unusual or have noteworthy features and alluded to the operational mode of action in the narrative. We consider that providing full mechanisms for each warhead would lead to a complicated and cluttered figure (or necessitate multiple figures).

'Figure 5: The overall illustration needs more clarity. The arrow pushing should initiate from the lone pair of the amine. The lemon-coloured component should be properly labeled to indicate its role in the system. In addition, the inclusion of UV irradiation for diazirine activation would provide mechanistic clarity.'

The curly arrows denoting the mechanism have been amended to ensure the arrow initiates from the amine lone pair, the limon component has been labelled 'PPI partner' and the need for UV irradiation for diazirine activation has been added to the reaction scheme.

'Page 10, line 248: The authors discuss KHAc, a derivative of a PGAM1 inhibitor, which may potentially alter the native activity of the target protein. Is there a pressing reason to keep this example, as it does not align with the arguments in the introduction?'

UNIVERSITY OF BIRMINGHAM

We have amended the discussion of KHAc to highlight that this is an example of t-LDL being applied for PTM rewiring. This example was included to showcase the application of t-LDL chemistry for inducing downstream effects. We have highlighted other examples elsewhere, including the PHICS developed by Pergu *et al.* (page 8-9), Singh *et* al's work on SuFA warheads for GRCs (page 14), and the synthetic histone acetylation developed by Kanai and coworkers (page 22-23). We consider the discussion of KHAc represents a notable example of chemistry used to achieve such effects will add value for the readers.

'Page 11, line 260: The authors state that "the SEAlide is unreactive in the absence of the protein target." What is the extent of selectivity? Does it imply exclusive selectivity towards the POI?'

We have expanded the discussion of SEALide reagents to highlight that N>S acyl transfer is observed with target protein *in vitro,* but not with small-molecule amines or cysteines. We have also used this section of the review to emphasise the importance of local protein environment to t-LDL chemistry and highlight challenges in characterising these chemistries.

'Page 11: The discussion related to reference 66 appears to involve a covalent inhibitor-based strategy. The authors should reconsider including this example or explain its relevance in the context of tracelessness.'

While reference 66 does report the application of the ArNASA warhead in the context of a covalent inhibitor, it is the first report of this warhead and details the improved reaction properties and stability relative to conventional NASA warheads. We feel this warrants its inclusion in the review but have updated the text to highlight that this initial application was for a covalent inhibitor.

'The authors discuss the first- and second-generation NASA-based LDL strategies; however, inclusion of a comparative figure illustrating the chemical modifications between these could help understand the structural changes that influence selectivity and reactivity.'

Figure 4 has been amended to include NASA and ArNASA as separate warheads to provide clarity.

'Figure 7c: The bonds are not drawn properly for iminoboronate.'

Figure 7c has been amended to correct the structure of the iminoboronate

'Figure 9: The authors primarily present the catalysts and reactive warheads; however, inclusion of the corresponding reaction mechanism would add clarity.'

We have included pertinent mechanistic details in Figures 10 and 9. As is the case with our response in relation to figure 3/4, we consider relevant mechanistic details are discussed in other more detailed figures and noted in the manuscript text. Adding full mechanisms here would lead to a complicated and cluttered diagram.

'Authors discuss the chemistry presented in references 102 and 116 in considerable detail; the inclusion of a representative figure/mechanism for these examples would add value.'

While we acknowledge the omission of a dedicated figure for AGD chemistry, we have included a representative example for the later developed and mechanistically similar AGOX chemistry. We consider that also including an AGD figure would be repetitive and there is more value added by illustrating the more recently introduced AGOC chemistry, which allows the use of NASA labelling reagents rather than thioesters and leading to more

UNIVERSITY OF BIRMINGHAM

effective/selective labelling. In lieu of a dedicated figure for AGD chemistry, we have amended the manuscript to note the mechanistic similarity of the two.

A similar rationale was applied in the case of the $[Ru(bpy)_3]^{2+}$ chemistry applied in reference 116 – Figure 11b features a representative example of $[Ru(bpy)_3]^{2+}$ mediated t-LDL and we are of the opinion that a separate figure would be unnecessarily duplicative.

'Other minor comments:'
'There are a few editing errors in the draft that require proofreading.'

We apologise for these typos and editing errors and have proofread the manuscript to correct these.

'The authors may consider numbering the subtitles for Lysine-targeting warheads as
4.2.1 Sulfonylation
4.2.2 Acylation'

We have added numbering to the subtitles to the lysine targeting warheads section to add clarity.

'Reference 90 doi: not available'

We have updated the reference to include the doi.

In summary, we hope these revisions are considered sufficient for our paper to be accepted for publication and look forward to hearing your response in due course.

Kind regards,

Andrew Wilson

Andy Wilson

Prof Andrew J Wilson PhD. BSc. (Hons) FRSC, FHEA
School of Chemistry

UNIVERSITY OF BIRMINGHAM

School of Chemistry
University of Birmingham
Edgbaston, Birmingham, B15 2TT, United Kingdom
a.j.wilson.1@bham.ac.uk

Date: 31st August 2026
Subject: COMMSCHEM-26-0366.R2

Dear Editor,

Please find attached a further revised review article, *"Progress and Challenges in Traceless Ligand-Directed Labelling Chemistry"* by Hopkinson, *et al*., which we hope is now considered acceptable for publication as a review in the Nature Portfolio Collection on *"Chemical modification of proteins"*, specifically *Communications Chemistry*. We are grateful to the reviewers for their comments and have addressed them with changes to the manuscript highlighted yellow (Figures 9 and 11 have also been revised). Our specific responses are as follows:

Reviewer #3

The authors state that a discussion of ligand-free protein labeling has been added to Section 1.1; however, I am unable to trace it in the revised manuscript.

In the original revision, we added a sentence in section 1.1 on reactive ligands (now marked in red). For this further revision, we reworded this sentence and added an additional sentence to contextualize this.

The C–S–C bond angle in Figure 3c appears to remain unchanged and still seems incorrect.

We apologize – we corrected two but not all three of these angles – this had now been addressed.

Figure 10b: Final product C–N–Carbonyl linkage - bond angle needs correction.

We have corrected this issue

The authors' response states that pertinent mechanistic details have been included in Figures 9 and 10; however, I do not see them in Figure 9 of the revised manuscript. The figure continues to present the catalysts and reactive warheads primarily, without indicating their corresponding modes of action.

There was an error in our cover letter: we meant to state that the pertinent mechanistic details are included in Figures 10 and 11. For this revision, we have now added additional detail of the SET mechanism in Figure 11, illustrating pathways for generation of a radical on the protein or trapping reagent respectively. To do so, we removed the structures of the μMap reagents and incorporated them into panel (d), and, reworded the entire caption accordingly. We have also stated the mechanistic class in Figure 9 (blue text highlighting nucleophilic catalysis of acylation for each of the organocatalysts, together with organometallic C-H insertion, single electron transfer (SET) and photocatalytic carbene activation followed by X-H insertion) to indicate the mode of action.

In summary, we hope these revisions are considered sufficient for our paper to be accepted for publication and look forward to hearing your response in due course.

Kind regards,

Andy Wilson

Prof Andrew J Wilson PhD, BSc. (Hons) FRSC, FHEA
School of Chemistry

UNIVERSITY OF BIRMINGHAM
